# Association of Healthful Plant-based Diet Adherence With Risk of Mortality and Major Chronic Diseases Among Adults in the UK

**DOI:** 10.1001/jamanetworkopen.2023.4714

**Published:** 2023-03-28

**Authors:** Alysha S. Thompson, Anna Tresserra-Rimbau, Nena Karavasiloglou, Amy Jennings, Marie Cantwell, Claire Hill, Aurora Perez-Cornago, Nicola P. Bondonno, Neil Murphy, Sabine Rohrmann, Aedín Cassidy, Tilman Kühn

**Affiliations:** 1 Institute for Global Food Security, School of Biological Sciences, Queen’s University, Belfast, United Kingdom; 2 Department of Nutrition, Food Science, and Gastronomy, School of Pharmacy and Food Sciences, Institute for Research on Nutrition and Food Safety, University of Barcelona, Barcelona, Spain; 3 Centro de Investigación Biomédica en Red Fisiopatología de la Obesidad y la Nutrición, Institute of Health Carlos III, Madrid, Spain; 4 Division of Chronic Disease Epidemiology, Epidemiology, Biostatistics, and Prevention Institute, University of Zurich, Zurich, Switzerland; 5 Centre for Public Health, Queen’s University, Belfast, United Kingdom; 6 Cancer Epidemiology Unit, Nuffield Department of Population Health, University of Oxford, Oxford, United Kingdom; 7 Danish Cancer Society Research Centre, Copenhagen, Denmark; 8 Nutrition and Health Innovation Research Institute, School of Medical and Health Sciences, Edith Cowan University, Joondalup, Western Australia, Australia; 9 Nutrition and Metabolism Branch, International Agency for Research on Cancer, Lyon, France; 10 Heidelberg Institute of Global Health, Faculty of Medicine and University Hospital, Heidelberg, Germany

## Abstract

**Importance:**

Plant-based diets have gained popularity for both environmental and health reasons, but a comprehensive assessment of their quality in relation to risk of mortality and major chronic diseases is lacking.

**Objective:**

To examine whether healthful vs unhealthful plant-based dietary patterns are associated with mortality and major chronic diseases among UK adults.

**Design, Setting, and Participants:**

This prospective cohort study used data from adults in the UK Biobank, a large-scale population-based study. Participants were recruited between 2006 and 2010 and followed up using record linkage data until 2021; follow-up for different outcomes ranged between 10.6 and 12.2 years. Data analysis was conducted from November 2021 to October 2022.

**Exposures:**

Adherence to a healthful vs unhealthful plant-based diet index (hPDI vs uPDI) derived from 24-hour dietary assessments.

**Main Outcomes and Measures:**

The main outcomes were hazard ratios (HRs) and 95% CIs of mortality (overall and cause specific), cardiovascular disease (CVD [total, myocardial infarction, ischemic stroke, and hemorrhagic stroke]), cancer (total, breast, prostate, and colorectal), and fracture (total, vertebrae, and hip) across quartiles of hPDI and uPDI adherence.

**Results:**

This study included 126 394 UK Biobank participants. They had a mean (SD) age of 56.1 (7.8) years; 70 618 (55.9%) were women. The majority of participants (115 371 [91.3%]) were White. Greater adherence to the hPDI was associated with lower risks of total mortality, cancer, and CVD, with HRs (95% CIs) of 0.84 (0.78-0.91), 0.93 (0.88-0.99), and 0.92 (0.86-0.99), respectively, for participants in the highest hPDI quartile compared with the lowest. The hPDI was also associated with lower risks of myocardial infarction and ischemic stroke, with HRs (95% CIs) of 0.86 (0.78-0.95) and 0.84 (0.71-0.99), respectively. By contrast, higher uPDI scores were associated with higher risks of mortality, CVD, and cancer. The associations observed did not show heterogeneity across strata of sex, smoking status, body mass index, or socioeconomic status or with polygenic risk scores (specifically with regard to CVD end points).

**Conclusions and Relevance:**

The findings of this cohort study of middle-aged UK adults suggest that a diet characterized by high-quality plant-based foods and lower intakes of animal products may be beneficial for health, irrespective of established chronic disease risk factors and genetic predisposition.

## Key Points

**Question:**

Is adherence to a healthful plant-based diet associated with a lower risk of mortality and chronic disease among UK adults?

**Findings:**

In this cohort study with 126 394 UK Biobank participants, greater adherence to a healthful plant-based diet was associated with a lower risk of mortality, cancer, and particularly cardiovascular disease. Opposing associations with higher risk were observed for individuals who adhered to an unhealthy plant-based diet.

**Meaning:**

The findings of this study suggest that a healthful plant-based diet that is low in animal foods, sugary drinks, snacks and desserts, refined grains, potatoes, and fruit juices was associated with a lower risk of mortality and major chronic diseases among adults in the UK.

## Teaser

This cohort study examines the association of healthful plant-based diet adherence with lower risk of mortality and major chronic diseases among UK adults.

## Introduction

Plant-based diets (PBDs) characterized by low consumption or complete omission of eggs, dairy products, fish, and meat are becoming increasingly popular worldwide.^1,2^ This trend is explained, in part, by potential health benefits among persons following a PBD, including a lower risk of diabetes, cardiovascular disease (CVD), and mortality.^3,4,5^ These diets are also being promoted for their favorable environmental footprint, as production of more plant foods and fewer animal foods may lead to reduced greenhouse gas emissions, fertilizer application, and land and freshwater use but greater biodiversity.^6,7,8^

Previous population-based studies have shown that PBDs per se may not be associated with improved health but that their composition is crucial with respect to a reduction in chronic disease risk.^6^ Only a healthful PBD, characterized by low consumption of both animal foods and processed foods of plant origin (eg, refined grains, sugary drinks, snacks and desserts) has been associated with a lower risk of type 2 diabetes,^9^ CVD,^5,10^ and total mortality.^3,11^ Lower risks of diabetes, ischemic heart disease, CVD mortality, and total mortality have also been observed among people following a strict vegan (omission of any animal foods) or vegetarian (omission of fish and meat) diet.^12,13^ However, strict PBDs may be associated with insufficient vitamin B_12_ and calcium intakes, the latter of which may increase the risk of osteoporosis and fracture.^14^ Moreover, despite increasing numbers of individuals consuming a vegan or vegetarian diet in many countries, less strict flexitarian PBDs that contain lower amounts of animal products may be adhered to more easily by many people compared with a vegan or vegetarian diet.^2^

Our objective was to examine the potential health benefits and risks associated with PBDs using data from the UK Biobank, a large-scale population-based study of UK adults, with a focus on diet quality. Specifically, we evaluated whether adherence to a healthful vs unhealthful type of PBD was associated with total and cause-specific mortality and with major chronic diseases (eg, CVD, cancer, and fracture, the latter particularly because higher fracture risk may be an adverse effect of PBDs).^14^

## Methods

### Study Population

The UK Biobank is a large-scale, population-based, prospective study consisting of more than 500 000 participants aged between 40 and 69 years at the time of recruitment between 2006 and 2010.^15^ Participants attended 1 of 22 assessment centers located across England, Scotland, and Wales, where they completed a comprehensive baseline assessment. Further details of the study protocol have been described elsewhere.^16^ The National Health Service (NHS) North West Multi-centre Research Ethics Committee approved the UK Biobank study. At recruitment, all participants provided written informed consent.

For this cohort study, participants who withdrew consent during follow-up, had missing data on diet or key covariates, had completed fewer than two 24-hour dietary assessments, or had implausible energy intakes (>17 573 or <3347 kJ for men and >14 644 or <2092 kJ for women^17^) were excluded from analyses. Additionally and depending on the outcome of interest, participants who had prevalent CVD, cancer, or fracture at recruitment or were diagnosed before completion of their last 24-hour dietary assessment were excluded from the analysis (eFigure 1 in Supplement 1). This study followed the Strengthening the Reporting of Observational Studies in Epidemiology (STROBE) reporting guideline.

### PBD Indices

In line with previous studies,^4,5,18^ the Oxford WebQ tool^19,20^ was used to construct a healthful plant-based diet index (hPDI) and an unhealthful plant-based diet index (uPDI), based on mean food intakes taken from a minimum of two 24-hour dietary assessments (eMethods 1 in Supplement 1). The plant-based diet indexes (PDIs) were made up of 17 food groups: whole grains, fruits, vegetables, nuts, legumes and vegetarian protein alternatives, tea and coffee, fruit juices, refined grains, potatoes, sugar-sweetened beverages, sweets and desserts, animal fat, dairy, eggs, fish or seafood, meat, and miscellaneous animal-derived foods.^20^ Data on vegetable oils, which were used to create the PDIs in previous studies, were not available.^18^ For the hPDI, healthy plant foods were scored positively and less healthy plant foods and animal-derived foods were scored negatively; the uPDI was scored oppositely to this (Table).^18^ Intakes of more than 0 portions were ranked into quartiles for each food group. Participants were ranked in quartiles for portions of each food group and were assigned a score between 2 and 5 (with 2 for the lowest category of intake and 5 for the highest). Participants with 0 intake were assigned a score of 1. A final hPDI and uPDI score for each participant was calculated by summing the scores of each of the 17 food groups and categorizing them into sex-specific quartiles.

**Table.** Baseline Characteristics Across Healthful Plant-based Diet Index Quartiles Among UK Biobank Participants

Characteristic	Value (n = 126 217)^a^			
	Quartile 1 (n = 33 901)	Quartile 2 (n = 30 427)	Quartile 3 (n = 30 007)	Quartile 4 (n = 31 882)
hPDI score, mean (SD)	47.7 (3.3)	53.7 (1.5)	57.7 (1.5)	63.4 (3.3)
Sex				
Women	18 082 (53.3)	17 072 (56.1)	17 143 (57.1)	18 258 (57.3)
Men	15 819 (46.7)	13 355 (43.9)	12 864 (42.9)	13 624 (42.7)
Age at recruitment, mean (SD), y	54.6 (8.1)	56.2 (7.8)	56.7 (7.6)	57.2 (7.4)
BMI, mean (SD)	27.7 (5.0)	26.8 (4.5)	26.4 (4.3)	25.8 (4.2)
Physical activity level				
Low	12 621 (37.2)	10 331 (34.0)	9479 (31.6)	8895 (27.9)
Moderate	10 618 (31.3)	10 004 (32.9)	9963 (33.2)	10 713 (33.6)
High	9963 (29.4)	9529 (31.3)	10 020 (33.4)	11 780 (37.0)
Race and ethnicity				
Asian	1405 (4.1)	1316 (4.3)	1423 (4.7)	1700 (5.3)
Black	113 (0.3)	104 (0.3)	108 (0.4)	141 (0.7)
Multiple	991 (2.9)	840 (2.8)	786 (2.6)	913 (2.9)
White	31 100 (91.7)	27 897 (91.7)	27 421 (91.4)	28 790 (90.3)
Other^b^	187 (0.6)	154 (0.5)	183 (0.6)	219 (0.7)
Education level				
Low	9321 (27.5)	7948 (26.1)	1950 (24.9)	7588 (23.8)
Medium	6039 (17.8)	5033 (16.5)	7469 (15.2)	4579 (14.4)
High	15 969 (47.1)	15 232 (50.1)	4570 (53.4)	17 860 (56.0)
Smoking status				
Never	19 462 (57.4)	17 310 (56.9)	17 174 (57.2)	18 157 (57.0)
Previous	11 392 (33.6)	10 892 (35.8)	10 859 (36.2)	11 979 (37.6)
Current	2984 (8.8)	2148 (7.1)	1905 (6.4)	1682 (5.3)
Alcohol intake, mean (SD), g/d	13.5 (19.4)	13.2 (18.1)	12.7 (17.8)	11.2 (17.0)
Medication use				
Aspirin	4405 (13.0)	3919 (12.9)	3941 (13.1)	4317 (13.5)
Multimorbidity, No. of long-term conditions				
0	13 833 (40.8)	12 642 (41.6)	12 417 (41.4)	13 482 (42.3)
1	10 248 (30.2)	9083 (29.9)	9223 (30.7)	9547 (29.9)
2	5589 (16.5)	5072 (16.7)	4980 (16.6)	5191 (16.3)
≥3	4231 (12.5)	3630 (11.9)	3387 (11.3)	3662 (11.5)
Polypharmacy, No. of medications				
0	10 281 (30.3)	9343 (30.7)	9402 (31.3)	10 148 (31.8)
1-3	15 889 (46.9)	14 262 (46.9)	13 935 (46.4)	14 765 (46.3)
4-6	5600 (16.5)	5021 (16.5)	4962 (16.5)	5186 (16.3)
7-9	1508 (4.5)	1310 (4.3)	1226 (4.2)	1367 (4.3)
≥10	619 (1.8)	481 (1.6)	438 (1.5)	413 (1.3)
hPDI food item intake, portion/d^c^				
Healthy plant food				
Whole grains	1.6 (1.3)	2.0 (1.4)	2.3 (1.4)	2.8 (1.5)
Fruit	1.5 (1.2)	2.0 (1.4)	2.5 (1.6)	3.2 (1.7)
Vegetables	1.7 (1.4)	2.2 (1.6)	2.6 (1.8)	3.5 (2.2)
Nuts	0.1 (0.2)	0.1 (0.3)	0.2 (0.3)	0.3 (0.5)
Legumes	0.3 (0.3)	0.3 (0.4)	0.4 (0.4)	0.6 (0.6)
Tea and coffee	3.9 (1.6)	4.2 (1.6)	4.5 (1.6)	4.9 (1.7)
Unhealthy plant food				
Refined grains	1.7 (1.2)	1.1 (1.0)	0.8 (0.8)	0.5 (0.6)
Potatoes	0.9 (0.6)	0.7 (0.5)	0.6 (0.5)	0.5 (0.5)
Sugary drinks	0.8 (1.0)	0.5 (0.8)	0.4 (0.6)	0.2 (0.5)
Fruit juices	0.6 (0.6)	0.5 (0.5)	0.4 (0.5)	0.3 (0.5)
Sweets and desserts	1.8 (1.3)	1.5 (1.2)	1.3 (1.1)	0.9 (1.0)
Animal fat	1.1 (1.2)	0.7 (1.0)	0.5 (0.9)	0.3 (0.7)
Dairy	1.2 (0.8)	1.1 (0.7)	1.1 (0.7)	1.0 (0.8)
Eggs	0.4 (0.5)	0.3 (0.4)	0.2 (0.4)	0.2 (0.4)
Fish or seafood	0.4 (0.4)	0.3 (0.4)	0.3 (0.4)	0.3 (0.4)
Meat	1.5 (0.9)	1.2 (0.8)	1.0 (0.8)	0.8 (0.7)
Miscellaneous animal-based foods	0.2 (0.4)	0.1 (0.3)	0.1 (0.2)	0 (0.2)

Abbreviations: BMI, body mass index (calculated as weight in kilograms divided by height in meters squared); hPDI, healthful plant-based diet index.

^a^ Relative frequencies (%) include missing values, which may not equate to 100%.

^b^ Other includes any racial and ethnic group not otherwise specified.

^c^ Portion sizes were specified as a “serving” in the Oxford WebQ tool.

### Covariate Assessment and Case Ascertainment

Baseline examinations of the UK Biobank cohort between 2006 and 2010 included questionnaire assessments of sociodemographic, dietary, and lifestyle factors. Anthropometric measurements and biological samples were collected from all study participants by trained staff.^16^ Further information on the covariates used in this study can be found in eMethods 2 in Supplement 1.

The case ascertainment in the UK Biobank cohort is also described in eMethods 3 in Supplement 1. In brief, data on mortality were available from the 2021 NHS death registries.^16^ For the CVD and fracture end points, hospital admission data were additionally available from the Hospital Episode Statistics for England (until September 2021), from Scottish Morbidity Records (until the end of July 2021), and from the Patient Episode Database for Wales (until March 2016). Cancer diagnosis data were provided through record linkage to National Cancer Registries in England, Wales (follow-up data available from the NHS Information Centre until February 2020), and Scotland (follow-up data available from the NHS Central Register of Scotland until January 2021).

### Statistical Analysis

Cox proportional hazards regression models with age as the underlying time variable were used to estimate hazard ratios (HRs) and 95% CIs of mortality (all-cause, CVD, or cancer), CVD (myocardial infarction, ischemic stroke, hemorrhagic stroke, or total), cancer (breast, prostate, colorectal, or total), and fracture risk (hip, vertebrae, or total) across PDI quartiles. Analyses were additionally adjusted for potential confounders, selected upon literature review. Further details on the main adjustments and missing values are provided in eMethods 4 and 5 in Supplement 1. Tests for linear trends were carried out by modeling the PDIs as continuous exposure variables (*P* for trend). *P* values from these tests were corrected for multiple testing by the Benjamini-Hochberg method (corrected *P*) to account for the fact that we had 2 exposures (hPDI and uPDI) and 14 end points. Cubic splines with knots at percentiles (5th, 35th, 65th, and 95th) were used to assess potential nonlinearity in associations between PDIs and end points. Sensitivity analyses were carried out, whereby participants with less than 2 years of follow-up (ie, 2 years after completion of their second 24-hour dietary assessment) were excluded from Cox proportional hazards regression models to account for potential reverse causality.

Subgroup analyses were carried out to assess potential heterogeneity in associations between PDIs and end points across strata of key confounders. These included sex, smoking status (ever or never), body mass index (BMI; <25 or ≥25 [calculated as weight in kilograms divided by height in meters squared]), and education level (low, medium, or high; eMethods 2 in Supplement 1). Moreover, heterogeneity by subgroups of polygenic risk score tertiles (low, medium, or high genetic risk) was assessed for outcomes for which data on polygenic risk scores were available from the UK Biobank (CVD or breast, bowel, or prostate cancer).^21^ The likelihood ratio test was used to test for interactions between PDIs and the covariates listed earlier in relation to mortality and disease end points, comparing the fits of Cox proportional hazards regression models with and without the respective interaction terms. We further carried out sensitivity analyses restricting the study sample to the majority of participants of White European ancestry, as both food preferences and disease risks may in part differ across races and ethnicities.^22,23^ Self-reported data on race and ethnicity for these analyses were obtained from the UK Biobank and categorized as Asian, Black, White, multiple races or ethnicities, and other race or ethnicity.

To test the reproducibility of the PDIs over time, we calculated intraclass coefficients (ICCs) between hPDI and uPDI values based on average food intakes at the second and third (collected in February-April 2011 and June-August 2011) vs the fourth and fifth (October-December 2011 and April-June 2012) dietary assessments via Oxford WebQ, as previously done for individual food groups in the UK Biobank.^24^ These analyses were based on a subgroup of 24 893 UK Biobank participants, who completed each of these 4 WebQ assessments.

All statistical analyses were conducted in Stata, version 17.0 (StataCorp LLC). Assessments of Schoenfeld residuals did not indicate violations of the proportional hazards assumption in any of our analyses. Associations were considered statistically significant at 2-sided *P* < .05. Data analysis was conducted from November 2021 to October 2022.

## Results

### Characteristics of the Study Population

Up to 126 394 of the 502 411 UK Biobank participants had data available from a minimum of 2 or more dietary recalls at baseline (mean [SD] completion, 2.9 [0.9]), including covariates relevant to this cohort study (eMethods 5 and eFigure 1 in Supplement 1). In this subsample, there were 70 618 women (55.9%) and 55 776 men (44.1%). Their mean (SD) age was 56.1 (7.8) years. A total of 5850 participants (4.6%) were Asian, 466 (0.4%) were Black, 115 371 (91.3%) were White, 3535 (2.8%) were of multiple races or ethnicities, and 744 (0.6%) and 428 (0.3%) were of other or unknown races or ethnicities, respectively. Baseline characteristics of the 126 394 included participants and 291 446 excluded participants vs all 502 411 participants in the UK Biobank cohort can be found in eTable 1 in Supplement 1. Depending on the end point being investigated, the size of sample subsets varied upon prevalent disease exclusion (eFigure 1 and eTable 2 in Supplement 1). Within a range of 10.6 to 12.2 years of follow-up for the different outcomes of interest in this study (eTable 2 in Supplement 1), there were 5627 deaths (698 attributable to CVD and 3275 to cancer), 6890 case patients with incident CVD (3253 with myocardial infarction, 1151 with ischemic stroke, and 469 with hemorrhagic stroke), 8939 case patients with incident cancer (1083 with postmenopausal breast cancer, 2137 with prostate cancer, and 959 with colorectal cancer), and 4751 case patients with incident fracture (736 with hip fracture and 319 with vertebrae fracture).

Both hPDI and uPDI scores were normally distributed across the study population, with ranges from 31 to 84 and from 28 to 82 points, respectively (eFigures 2 and 3 in Supplement 1). Baseline characteristics, including key nutrient intakes across hPDI and uPDI quartiles, are presented in the Table and eTables 3 to 5 in Supplement 1. Participants with higher hPDI scores were more likely to be female, to have a lower BMI, to be older, to report taking no medications, to report having no long-term health conditions, to have lower alcohol intakes at recruitment, and to have a high education level compared with participants with lower hPDI scores. The ICCs (ranges) for reproducibility of the PDI scores over time were 0.58 (34-83) for hPDI and 0.55 (29-77) for uPDI (eTable 6 in Supplement 1).

### PBDs and Mortality Risk

**Figure 1.**
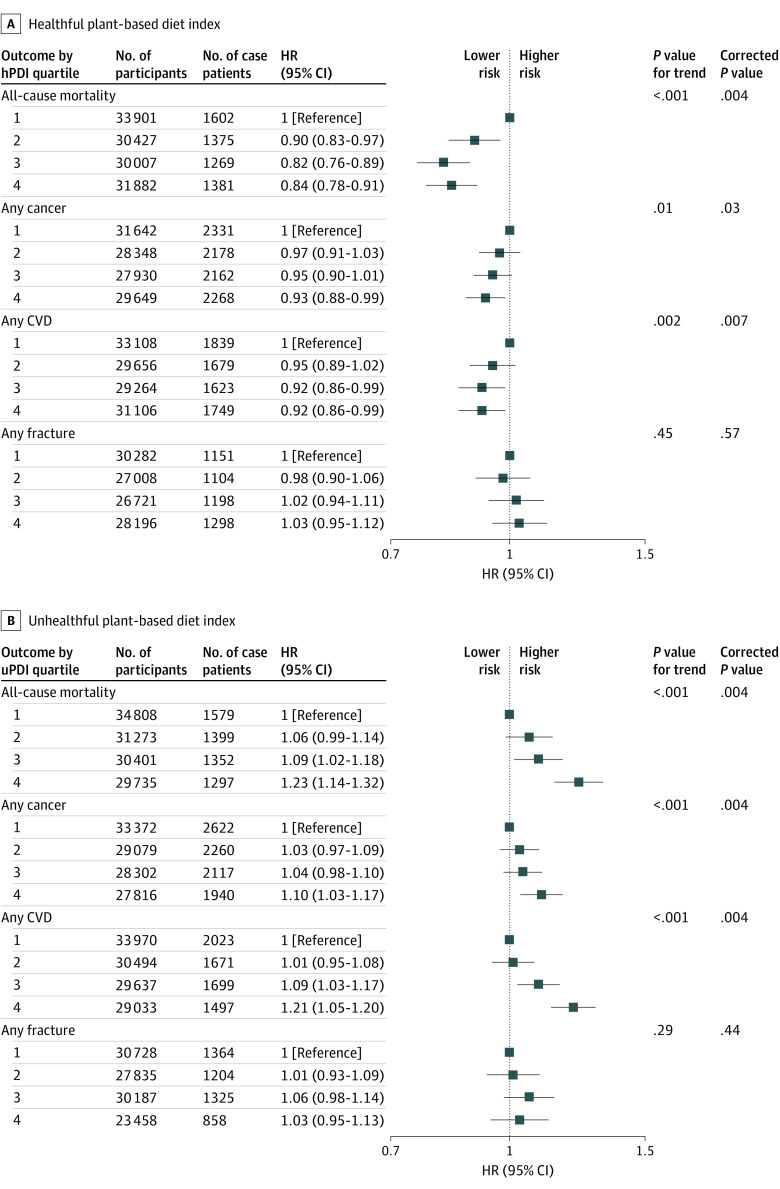
Multivariable-Adjusted Hazard Ratios and 95% CIs for All-Cause Mortality (n = 126 217), Cancer (n = 117 569), Cardiovascular Disease (CVD; n = 123 134), and Fracture (n = 112 208) Across Sex-Specific Healthful vs Unhealthful Plant-based Diet Index Quartiles All models used age as the underlying time variable and were adjusted for sex, body mass index, race and ethnicity, physical activity level, smoking status, alcohol intake, education level, energy intake, polypharmacy index, multimorbidity index, and aspirin use, stratified by region. For all-cause mortality analyses, models were further adjusted for prevalent CVD and prevalent cancer. For any cancer analyses, models were further adjusted for menopausal status and use of menopause hormone therapy. For any CVD analyses, models were further adjusted for polygenic risk score. For any fracture analyses, models were further adjusted for vitamin or mineral supplement use and polygenic risk score (osteoporosis). hPDI indicates healthful plant-based diet index; HR, hazard ratio; uPDI, unhealthful plant-based diet index.

In multivariable-adjusted models, participants with higher hPDI scores (quartile 4) compared with those with lower scores (quartile 1) had a 16% lower risk of all-cause mortality (HR, 0.84 [95% CI, 0.78-0.91]; corrected *P* = .004) (Figure 1 and eTable 7 in Supplement 1). By contrast, participants with higher uPDI scores had a 23% higher risk of all-cause mortality (HR, 1.23 [95% CI, 1.14-1.32]; corrected *P* = .004) (Figure 1 and eTable 8 in Supplement 1). With regard to cause-specific mortality, an association between uPDI and cancer mortality was observed (HR, 1.19 [95% CI, 1.08-1.32]; corrected *P* = .004) (eTables 7 and 8 in Supplement 1).

### PBDs and Cancer Risks

**Figure 2.**
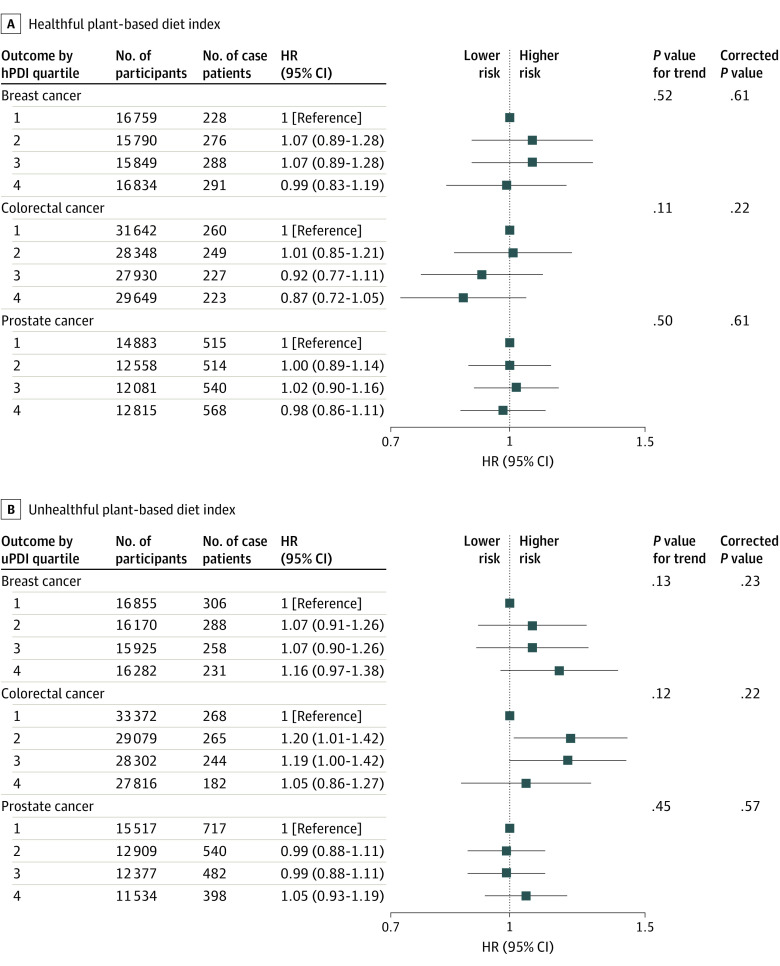
Multivariable-Adjusted Hazard Ratios and 95% CIs for Postmenopausal Breast Cancer, Colorectal Cancer, and Prostate Cancer Across Sex-Specific Healthful vs Unhealthful Plant-based Diet Index Quartiles (n = 117 569) All models used age as the underlying time variable and were adjusted for sex (excluding breast and prostate cancer), body mass index, race and ethnicity, physical activity level, smoking status, alcohol intake, education level, energy intake, polypharmacy index, multimorbidity index, and aspirin use, stratified by region. For breast cancer analyses, models were restricted to postmenopausal case patients with breast cancer and were further adjusted for use of menopause hormone therapy, use of oral contraception, polygenic risk score, age at menarche, and age at first live birth. For colorectal cancer analyses, models were further adjusted for menopausal status, polygenic risk score, and menopause hormone therapy. For prostate cancer analyses, models were further adjusted for polygenic risk score. hPDI indicates healthful plant-based diet index; HR, hazard ratio; uPDI, unhealthful plant-based diet index.

Greater hPDI adherence was associated with a 7% lower risk of any cancer (HR, 0.93 [95% CI, 0.88-0.99]; corrected *P* = .03) (Figure 1 and eTable 9 in Supplement 1), whereas participants with higher uPDI scores had a 10% higher risk (HR, 1.10 [95% CI, 1.03-1.17]; corrected *P* = .004) (Figure 1 and eTable 10 in Supplement 1). There were no associations between hPDI or uPDI and the most frequently diagnosed individual cancers (eg, breast, prostate, and colorectal cancer; Figure 2).

### PBDs and CVD

**Figure 3.**
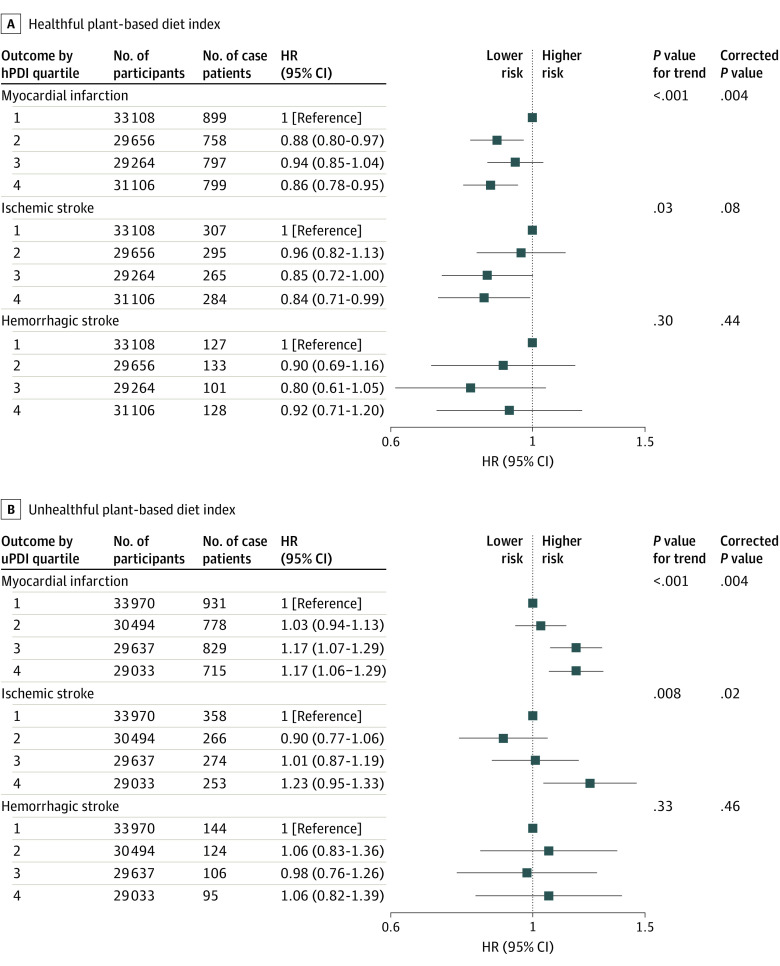
Multivariable-Adjusted Hazard Ratios and 95% CIs for Myocardial Infarction, Ischemic Stroke, and Hemorrhagic Stroke Across Sex-Specific Healthful vs Unhealthful Plant-based Diet Index Quartiles (n = 123 134) All models used age as the underlying time variable and were adjusted for sex, body mass index, race and ethnicity, physical activity level, smoking status, alcohol intake, education level, energy intake, polypharmacy index, multimorbidity index, and aspirin use, stratified by region. For myocardial infarction analyses, models were further adjusted for polygenic risk score (coronary artery disease). For ischemic stroke analyses, models were further adjusted for polygenic risk score (ischemic stroke). For hemorrhagic stroke analyses, models were further adjusted for polygenic risk score (cardiovascular disease). hPDI indicates healthful plant-based diet index; HR, hazard ratio; uPDI, unhealthful plant-based diet index.

Higher hPDI scores were associated with lower risks of total CVD, ischemic stroke, and myocardial infarction, with HRs (95% CIs) of 0.92 (0.86-0.99; corrected *P* = .007), 0.84 (0.71-0.99; corrected *P* = .08), and 0.86 (0.78-0.95; corrected *P* = .004) in multivariable models. Higher uPDI scores were associated with higher risks of total CVD, ischemic stroke, and myocardial infarction, with HRs (95% CIs) of 1.21 (1.05-1.20; corrected *P* = .004), 1.23 (0.95-1.33; corrected *P* = .02), and 1.17 (1.06-1.29; corrected *P* = .004) (Figures 1 and 3; and eTables 11 and 12 in Supplement 1). No associations between hPDI or uPDI and risk of hemorrhagic stroke were observed (Figure 3).

### PBDs and Fracture Risk

Our analyses did not show associations between hPDI or uPDI with risks of site-specific and total fracture. The findings of these analyses are provided in eTables 13 and 14 in Supplement 1.

### Subgroup and Sensitivity Analyses

**Figure 4.**
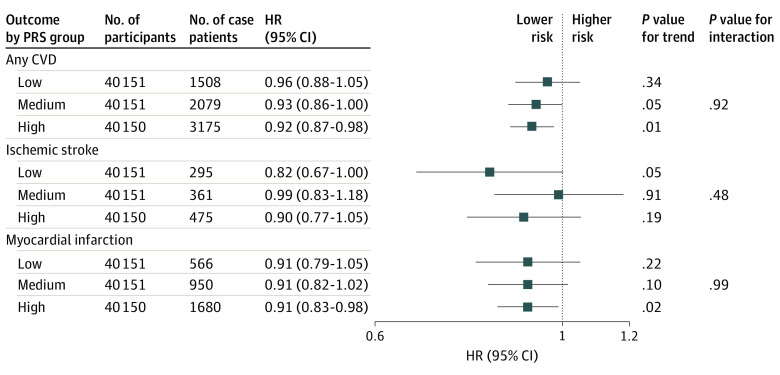
Multivariable-Adjusted Hazard Ratios and 95% CIs for Cardiovascular Disease (CVD) Across Strata of Genetic CVD, Ischemic Stroke, or Coronary Artery Disease Risk, With Healthful Plant-based Diet Index (hPDI) Score Modeled as a Continuous Trend (10-Point Increments) Analyses used age as the underlying time variable and were adjusted for sex, body mass index, race and ethnicity, physical activity level, smoking status, alcohol intake, education level, energy intake, polypharmacy index, multimorbidity index, and aspirin use, stratified by region. Heterogeneity was tested by comparing 2 models: one model without an interaction term between the subgroup of interest and hPDI compared with another model with an interaction term between the subgroup of interest and hPDI. The likelihood ratio test was used to produce *P* interaction values. HR indicates hazard ratio; PRS, polygenic risk score.

There was no indication for nonlinearity in associations between hPDI and mortality, cancer, or CVD (eFigure 4 in Supplement 1). Subgroup analyses did not indicate differential associations between hPDI and end points across strata of key covariates including polygenic risk scores (Figure 4 and eFigures 5-10 in Supplement 1), with very few exceptions. An inverse association between hPDI and CVD mortality was observed among ever smokers (HR, 0.77 [95% CI, 0.65-0.91]; *P* = .002 for trend) but not among nonsmokers (HR, 1.02, [95% CI, 0.84-1.24]; *P* = .85 for trend; eFigure 5 in Supplement 1). In addition, an inverse association between hPDI and ischemic stroke risk was observed among participants with a high education level (HR, 0.87 [95% CI, 0.75-1.00]; *P* = .06 for trend), but not among those with a low education level (HR, 0.98, [95% CI, 0.83-1.15]; *P* = .77 for trend; eFigure 7 in Supplement 1). When participants who had an event or were censored in the first 2 years of follow-up were excluded, results from Cox proportional hazards regression analyses on all end points remained similar, although the association between uPDI and breast cancer became statistically significant (eFigures 11-14 in Supplement 1). When stratifying analyses by ancestry, results for all-cause mortality, any cancer, any CVD, and any fracture remained highly similar for participants of White European ancestry. Inverse associations were observed between hPDI and uPDI for the same end points among groups with non-European ancestry. No significant differences were observed between White European and non-European populations for risk of all-cause mortality, any cancer, any CVD, and any fracture with hPDI and uPDI (eFigure 15 in Supplement 1).

## Discussion

In this cohort study, our prospective analyses of UK Biobank data showed that greater adherence to a healthful PBD was associated with lower risk of total mortality, while greater adherence to an unhealthful PBD was associated with a higher risk of mortality. This pattern of opposing associations was also observed with regard to incident CVD and cancer. In this study, adherence to a healthful or unhealthful PBD was not associated with fracture risk. Overall, our results suggest that a healthful plant-based dietary pattern—characterized by lower amounts of animal foods, sugary drinks, snacks and desserts, refined grains, potatoes, and fruit juices—is associated with lower risks of mortality and major chronic diseases.

Our findings are generally in line with those from previous US studies on hPDI and uPDI in relation to myocardial infarction, ischemic stroke, CVD mortality, and total mortality.^25,26,27^ Importantly, we observed that inverse associations between the hPDI and CVD end points (total CVD, myocardial infarction, and stroke) were independent of genetic disease risk. This finding is of particular public health relevance, as it suggests individual benefits of healthful PBDs irrespective of genetic predisposition for CVD. At the same time, absolute risk reduction due to a healthful PBD may be greater among individuals with a stronger genetic predisposition for CVD, given their higher lifetime risk of CVD.^28^

In contrast with our study that showed no association between hPDI and colorectal cancer risk, participants from 2 US cohorts with a higher hPDI had a significantly lower risk of colon cancer.^29^ However, point estimates for extreme quintile comparisons of 0.87 in our study and 0.86 in the US cohorts are similar, and the wider CIs in our study are likely due to the lower number of case patients (959 in the UK Biobank vs 3077 in the US cohorts). Unlike in our study where we observed no association, hPDI was associated with lower breast cancer risk in the Nurses’ Health Study^30^; the reason for this discrepancy remains unclear. Of note, ours and the aforementioned studies on PDI and major chronic diseases were carried out among predominantly White populations. One recent smaller study among Black individuals in the US did not show associations between hPDI or uPDI and mortality risks.^31^ Thus, future studies with wider racial and ethnic diversity are required.

Several mechanisms may underlie the associations between hPDI and lower risks of disease and mortality observed in our study. Higher consumption of unprocessed plant foods may reduce the risk of obesity, low-grade inflammation, and impaired insulin sensitivity.^6,32^ These mechanisms may explain lower mortality risks due to both CVD and cancer.^33^ Similarly, plant constituents such as fiber may beneficially affect the composition and function of the large intestinal microbiome, and bacterial metabolites such as short-chained fatty acids, bile acids, or trimethylamine-oxide may be associated with CVD and certain cancers, although further research in human populations is needed.^32,34,35,36^ The fact that associations between hPDI and CVD revealed lower risks, especially of myocardial infarction and ischemic stroke compared with that for cancer in our study, suggests that additional CVD-specific mechanisms (eg, lower blood pressure or low-density lipoprotein cholesterol due to PBD^6^) further explain our findings.

Plant-based diets are considered to be beneficial for planetary health.^7,37^ In addition, healthful PBDs are largely compatible with dietary recommendations for the prevention of chronic diseases across the globe,^38^ including those for a planetary health diet by the EAT-Lancet Commission on Food, Planet, Health.^39^ Our results provide further evidence to substantiate that PBD quality may be essential for individual health—that is, that PBDs are not beneficial per se and can even be detrimental to health depending on their composition. Interestingly, although we observed that the hPDI was associated with a lower risk of mortality, CVD, and cancer, we found no associations with hemorrhagic stroke and fracture. The latter have been reported to be more common among participants in the European Prospective Investigation Into Cancer and Nutrition (EPIC)-Oxford cohort who consumed a vegetarian or vegan diet,^40,41^ who have otherwise lower cardiometabolic disease risk.^42^ Our findings suggest that a healthful flexitarian type of PBD including lower amounts of animal foods may protect against such potential adverse effects of vegan or vegetarian diets. However, it should be acknowledged that the evidence on vegan and vegetarian diets in relation to hemorrhagic stroke risk is limited to the EPIC-Oxford study and a very small study from Taiwan, in which lower risk among vegetarians was observed.^43^ Moreover, higher fracture risk among individuals who follow a vegan or vegetarian diet can be addressed by sufficient intakes of critical nutrients, especially calcium, from nonanimal food sources.^44^

To our knowledge, this cohort study provides the first integrated analyses on PBDs in relation to mortality and major chronic diseases in a population-based prospective study, with a focus on both potential risks and benefits. The large numbers of case patients facilitated subgroup analyses across strata of key covariates, including polygenic risk scores.

### Limitations

This study has some limitations. Although the associations between PDI scores and end points showed very little heterogeneity across strata of potential confounders, we cannot rule out residual confounding. Another limitation is that we relied on at least two 24-hour dietary assessments to quantify habitual diet composition, although the consistency of our findings with those from cohorts based on repeated dietary assessments by food frequency questionnaires is striking.^3,5,10^ In turn, it can be assumed that we underestimated associations between PDIs and end points due to regression dilution, as indicated by the ICCs of 0.58 and 0.55 for repeated hPDI and uPDI assessments in our study. In addition, reasonable validity of the Oxford WebQ tool was shown in a recent biomarker study.^45^ Although the UK Biobank is a large prospective cohort study, its population is not representative of the general UK population, which may limit the generalizability of our findings.^46^ More than 90% of UK Biobank participants are White and of European ancestry. Our subgroup analyses among participants of non-European ancestry were based on a smaller sample size, which is why other studies on PBDs and health outcomes among people with different cultural, racial, and ethnic backgrounds are needed.

## Conclusions

The findings of this cohort study of 126 394 middle-aged adults from the UK suggest that a healthful PBD was associated with lower risks of CVD, cancer, and total mortality. On the contrary, a plant-based dietary pattern characterized by higher intakes of sugary drinks, snacks and desserts, refined grains, potatoes, and fruit juices was associated with higher risk. Our results support a shift toward food intake that emphasizes healthy plant foods to improve health and provide data to support a healthful PBD for CVD prevention irrespective of genetic disease risk. However, future studies among more racially, ethnically, and culturally diverse populations are needed to assess the risk of major chronic disease in relation to PBDs.

## Notes

Supplement 1. **eMethods 1.** Dietary Intake Assessment **eMethods 2.** Covariate Information **eMethods 3.** Outcome Ascertainment **eMethods 4.** Multivariable Adjustment **eMethods 5.** Missing Values **eTable 1. **Comparison of Baseline Characteristics of the UK Biobank Cohort (n = 502 411), Included Participants (n = 126 394), and Excluded Participants (n = 291 446) **eTable 2.** Cases, Follow-up Years, and Number Lost to Follow-up for All-Cause Mortality and Incident Cancer, Cardiovascular Disease, and Fracture Outcomes **eTable 3.** Key Nutrient Intakes Across Healthful Plant-based Diet Index Quartiles (n = 126 217) **eTable 4.** Food Groups and Key Nutrient Intakes Across Unhealthful Plant-based Diet Index Quartiles (n = 126 217) **eTable 5.** Baseline Characteristics Across Unhealthful Plant-based Diet Index Quartiles (n = 126 217) **eTable 6.** Intraclass Correlation Coefficients for Reproducibility of Plant-based Diet Index Scores Over Time **eTable 7.** Hazard Ratios (95% CIs) of Mortality Across Sex-Specific Healthful Plant-based Diet Index Quartiles (n = 126 217) **eTable 8.** Hazard Ratios (95% CIs) of Mortality Across Sex-Specific Unhealthful Plant-based Diet Index Quartiles (n = 126 217) **eTable 9.** Hazard Ratios (95% CIs) of Cancer (Postmenopausal Breast, Colorectal, Prostate, or Any) Across Sex-Specific Healthful Plant-based Diet Index Quartiles (n = 117 569) **eTable 10.** Hazard Ratios (95% CIs) of Cancer (Postmenopausal Breast, Colorectal, Prostate, or Any) Across Sex-Specific Unhealthful Plant-based Diet Index Quartiles (n = 117 569) **eTable 11.** Hazard Ratios (95% CIs) of Cardiovascular Disease (Myocardial Infarction, Stroke, or Any) Across Sex-Specific Healthful Plant-based Diet Index Quartiles (n = 123 134) **eTable 12.** Hazard Ratios (95% CIs) of Cardiovascular Disease (Myocardial Infarction, Stroke, or Any) Across Sex-Specific Unhealthful Plant-based Diet Index Quartiles (n = 123 134) **eTable 13.** Hazard Ratios (95% CIs) of Fracture (Hip, Vertebrae, or Any) Across Sex-Specific Healthful Plant-based Diet Index Quartiles (n = 112 208) **eTable 14.** Hazard Ratios (95% CIs) of Fracture (Hip, Vertebrae, or Any) Across Sex-Specific Unhealthful Plant-based Diet Index Quartiles (n = 112 208) **eFigure 1.** Flow Diagram of UK Biobank Participants Excluded From Analysis **eFigure 2.** Histogram Showing Total Healthful Plant-based Diet Index (hPDI) Scores of UK Biobank Participants (n = 126 217) **eFigure 3.** Histogram Showing Total Unhealthful Plant-based Diet Index (uPDI) Scores of UK Biobank Participants (n = 126 217) **eFigure 4.** Cubic Spline Graphs Showing Fully Adjusted Linear Associations of Healthful Plant-based Diet Index (hPDI) and Unhealthful Plant-based Diet Index (uPDI) Scores With All-Cause Mortality, Cancer, Cardiovascular Disease (CVD), and Fracture Risk **eFigure 5.** Hazard Ratios (95% CIs) of All-Cause, Cancer, and Cardiovascular Disease Mortality Among UK Biobank Population Subgroups, With Healthful Plant-based Diet Score Modeled as a Continuous Trend (10-Point Increments) **eFigure 6.** Hazard Ratios (95% CIs) of Cancer Among UK Biobank Subgroups, With Healthful Plant-based Diet Score Modeled as a Continuous Trend (10-Point Increments) **eFigure 7.** Hazard Ratios (95% CIs) of Cardiovascular Disease Among UK Biobank Subgroups, With Healthful Plant-based Diet Score Modeled as a Continuous Trend (10-Point Increments) **eFigure 8.** Hazard Ratios (95% CIs) of Fracture Among UK Biobank Subgroups, With Healthful Plant-based Diet Score Modeled as a Continuous Trend (10-Point Increments) **eFigure 9.** Hazard Ratios (95% CIs) of Cancer Across Strata of Genetic Cancer Risk, With Healthful Plant-based Diet Score Modeled as a Continuous Trend (10-Point Increments) **eFigure 10.** Hazard Ratios (95% CIs) of Fracture Across Strata of Genetic Osteoporosis Risk, With Healthful Plant-based Diet Score Modeled as a Continuous Trend (10-Point Increments) **eFigure 11.** Sensitivity Analyses Showing Hazard Ratios (95% CIs) Across Sex-Specific Healthful vs Unhealthful Plant-based Diet Index Quartiles, Removing the First 2 Years of Follow-up for Participants Who Completed 2 or More Dietary Assessments and the Associated Risk of All-Cause, Cancer, and Cardiovascular Disease Mortality **eFigure 12.** Sensitivity Analyses Showing Hazard Ratios (95% CIs) Across Sex-Specific Healthful vs Unhealthful Plant-based Diet Index Quartiles, Removing the First 2 Years of Follow-up for Participants Who Completed 2 or More Dietary Assessments and the Associated Risk of Any Cancer, Postmenopausal Breast Cancer, Colorectal Cancer, and Prostate Cancer **eFigure 13.** Sensitivity Analyses Showing Hazard Ratios (95% CIs) Across Sex-Specific Healthful vs Unhealthful Plant-based Diet Index Quartiles, Removing the First 2 Years of Follow-up for Participants Who Completed 2 or More Dietary Assessments and the Associated Risk of Any Cardiovascular Disease, Ischemic Stroke, Hemorrhagic Stroke, and Myocardial Infarction **eFigure 14.** Sensitivity Analyses Showing Hazard Ratios (95% CIs) Across Sex-Specific Healthful vs Unhealthful Plant-based Diet Index Quartiles, Removing the First 2 Years of Follow-up for Participants Who Completed 2 or More Dietary Assessments and the Associated Risk of Any Fracture, Hip Fracture, and Vertebrae Fracture **eFigure 15.** Multivariable-Adjusted Hazard Ratios (95% CIs) of All-Cause Mortality (n = 126 217), Cancer (n = 117 569), Cardiovascular Disease (n = 123 134), and Fracture (n = 112 208) Among Racial and Ethnic Subgroups Across Sex-Specific Healthful vs Unhealthful Plant-based Diet Index Quartiles
eReferences

Supplement 2.
Data Sharing Statement
