# Supplementary material for: Association of Healthful Plant-based Diet Adherence With Risk of Mortality and Major Chronic Diseases Among Adults in the UK
Source: JAMA Netw Open. 2023 Mar 28;6(3):e234714. doi: 10.1001/jamanetworkopen.2023.4714 (PMC10051114; doi:10.1001/jamanetworkopen.2023.4714)
Supplement: Supplement 1. — eMethods 1. Dietary Intake Assessment eMethods 2. Covariate Information eMethods 3. Outcome Ascertainment eMethods 4. Multivariable Adjustment eMethods 5. Missing Values eTable 1. Comparison of Baseline Characteristics of the UK Biobank Cohort (n = 502 411), Included Participants (n = 126 394), and Excluded Participants (n = 291 446) eTable 2. Cases, Follow-up Years, and Number Lost to Follow-up for All-Cause Mortality and Incident Cancer, Cardiovascular Disease, and Fracture Outcomes eTable 3. Key Nutrient Intakes Across Healthful Plant-based Diet Index Quartiles (n = 126 217) eTable 4. Food Groups and Key Nutrient Intakes Across Unhealthful Plant-based Diet Index Quartiles (n = 126 217) eTable 5. Baseline Characteristics Across Unhealthful Plant-based Diet Index Quartiles (n = 126 217) eTable 6. Intraclass Correlation Coefficients for Reproducibility of Plant-based Diet Index Scores Over Time eTable 7. Hazard Ratios (95% CIs) of Mortality Across Sex-Specific Healthful Plant-based Diet Index Quartiles (n = 126 217) eTable 8. Hazard Ratios (95% CIs) of Mortality Across Sex-Specific Unhealthful Plant-based Diet Index Quartiles (n = 126 217) eTable 9. Hazard Ratios (95% CIs) of Cancer (Postmenopausal Breast, Colorectal, Prostate, or Any) Across Sex-Specific Healthful Plant-based Diet Index Quartiles (n = 117 569) eTable 10. Hazard Ratios (95% CIs) of Cancer (Postmenopausal Breast, Colorectal, Prostate, or Any) Across Sex-Specific Unhealthful Plant-based Diet Index Quartiles (n = 117 569) eTable 11. Hazard Ratios (95% CIs) of Cardiovascular Disease (Myocardial Infarction, Stroke, or Any) Across Sex-Specific Healthful Plant-based Diet Index Quartiles (n = 123 134) eTable 12. Hazard Ratios (95% CIs) of Cardiovascular Disease (Myocardial Infarction, Stroke, or Any) Across Sex-Specific Unhealthful Plant-based Diet Index Quartiles (n = 123 134) eTable 13. Hazard Ratios (95% CIs) of Fracture (Hip, Vertebrae, or Any) Across Sex-Specific Healthful Plant-based Diet Index Quarti [file jamanetwopen-e234714-s001.pdf]

Supplementary Online Content

Thompson AS, Tresserra-Rimbau A, Karavasiloglou N, et al. Association of healthful plant-based diet adherence with risk of mortality and major chronic diseases among adults in the UK. *JAMA Netw Open*. 2023;6(3):e234714. doi:10.1001/jamanetworkopen.2023.4714

eMethods 1. Dietary Intake Assessment

eMethods 2. Covariate Information

eMethods 3. Outcome Ascertainment

eMethods 4. Multivariable Adjustment

eMethods 5. Missing Values

eTable 1. Comparison of Baseline Characteristics of the UK Biobank Cohort (n = 502 411), Included Participants (n = 126 394), and Excluded Participants (n = 291 446)

eTable 2. Cases, Follow-up Years, and Number Lost to Follow-up for All-Cause Mortality and Incident Cancer, Cardiovascular Disease, and Fracture Outcomes

eTable 3. Key Nutrient Intakes Across Healthful Plant-based Diet Index Quartiles (n = 126 217)

eTable 4. Food Groups and Key Nutrient Intakes Across Unhealthful Plant-based Diet Index Quartiles (n = 126 217)

eTable 5. Baseline Characteristics Across Unhealthful Plant-based Diet Index Quartiles (n = 126 217)

eTable 6. Intraclass Correlation Coefficients for Reproducibility of Plant-based Diet Index Scores Over Time

eTable 7. Hazard Ratios (95% CIs) of Mortality Across Sex-Specific Healthful Plant-based Diet Index Quartiles (n = 126 217)

eTable 8. Hazard Ratios (95% CIs) of Mortality Across Sex-Specific Unhealthful Plant-based Diet Index Quartiles (n = 126 217)

eTable 9. Hazard Ratios (95% CIs) of Cancer (Postmenopausal Breast, Colorectal, Prostate, or Any) Across Sex-Specific Healthful Plant-based Diet Index Quartiles (n = 117 569)

eTable 10. Hazard Ratios (95% CIs) of Cancer (Postmenopausal Breast, Colorectal, Prostate, or Any) Across Sex-Specific Unhealthful Plant-based Diet Index Quartiles (n = 117 569)

eTable 11. Hazard Ratios (95% CIs) of Cardiovascular Disease (Myocardial Infarction, Stroke, or Any) Across Sex-Specific Healthful Plant-based Diet Index Quartiles (n = 123 134)

eTable 12. Hazard Ratios (95% CIs) of Cardiovascular Disease (Myocardial Infarction, Stroke, or Any) Across Sex-Specific Unhealthful Plant-based Diet Index Quartiles (n = 123 134)

eTable 13. Hazard Ratios (95% CIs) of Fracture (Hip, Vertebrae, or Any) Across Sex-Specific Healthful Plant-based Diet Index Quartiles (n = 112 208)

eTable 14. Hazard Ratios (95% CIs) of Fracture (Hip, Vertebrae, or Any) Across Sex-Specific Unhealthful Plant-based Diet Index Quartiles (n = 112 208)

eFigure 1. Flow Diagram of UK Biobank Participants Excluded From Analysis

eFigure 2. Histogram Showing Total Healthful Plant-based Diet Index (hPDI) Scores of UK Biobank Participants (n = 126 217)

© 2023 Thompson AS et al. *JAMA Network Open*.

eFigure 3. Histogram Showing Total Unhealthful Plant-based Diet Index (uPDI) Scores of UK Biobank Participants (n = 126 217)

eFigure 4. Cubic Spline Graphs Showing Fully Adjusted Linear Associations of Healthful Plant-based Diet Index (hPDI) and Unhealthful Plant-based Diet Index (uPDI) Scores With All-Cause Mortality, Cancer, Cardiovascular Disease (CVD), and Fracture Risk

eFigure 5. Hazard Ratios (95% CIs) of All-Cause, Cancer, and Cardiovascular Disease Mortality Among UK Biobank Population Subgroups, With Healthful Plant-based Diet Score Modeled as a Continuous Trend (10-Point Increments)

eFigure 6. Hazard Ratios (95% CIs) of Cancer Among UK Biobank Subgroups, With Healthful Plant-based Diet Score Modeled as a Continuous Trend (10-Point Increments)

eFigure 7. Hazard Ratios (95% CIs) of Cardiovascular Disease Among UK Biobank Subgroups, With Healthful Plant-based Diet Score Modeled as a Continuous Trend (10-Point Increments)

eFigure 8. Hazard Ratios (95% CIs) of Fracture Among UK Biobank Subgroups, With Healthful Plant-based Diet Score Modeled as a Continuous Trend (10-Point Increments)

eFigure 9. Hazard Ratios (95% CIs) of Cancer Across Strata of Genetic Cancer Risk, With Healthful Plant-based Diet Score Modeled as a Continuous Trend (10-Point Increments)

eFigure 10. Hazard Ratios (95% CIs) of Fracture Across Strata of Genetic Osteoporosis Risk, With Healthful Plant-based Diet Score Modeled as a Continuous Trend (10-Point Increments)

eFigure 11. Sensitivity Analyses Showing Hazard Ratios (95% CIs) Across Sex-Specific Healthful vs Unhealthful Plant-based Diet Index Quartiles, Removing the First 2 Years of Follow-up for Participants Who Completed 2 or More Dietary Assessments and the Associated Risk of All-Cause, Cancer, and Cardiovascular Disease Mortality

eFigure 12. Sensitivity Analyses Showing Hazard Ratios (95% CIs) Across Sex-Specific Healthful vs Unhealthful Plant-based Diet Index Quartiles, Removing the First 2 Years of Follow-up for Participants Who Completed 2 or More Dietary Assessments and the Associated Risk of Any Cancer, Postmenopausal Breast Cancer, Colorectal Cancer, and Prostate Cancer

eFigure 13. Sensitivity Analyses Showing Hazard Ratios (95% CIs) Across Sex-Specific Healthful vs Unhealthful Plant-based Diet Index Quartiles, Removing the First 2 Years of Follow-up for Participants Who Completed 2 or More Dietary Assessments and the Associated Risk of Any Cardiovascular Disease, Ischemic Stroke, Hemorrhagic Stroke, and Myocardial Infarction

eFigure 14. Sensitivity Analyses Showing Hazard Ratios (95% CIs) Across Sex-Specific Healthful vs Unhealthful Plant-based Diet Index Quartiles, Removing the First 2 Years of Follow-up for Participants Who Completed 2 or More Dietary Assessments and the Associated Risk of Any Fracture, Hip Fracture, and Vertebrae Fracture

eFigure 15. Multivariable-Adjusted Hazard Ratios (95% CIs) of All-Cause Mortality (n = 126 217), Cancer (n = 117 569), Cardiovascular Disease (n = 123 134), and Fracture (n = 112 208) Among Racial and Ethnic Subgroups Across Sex-Specific Healthful vs Unhealthful Plant-based Diet Index Quartiles

eReferences

This supplementary material has been provided by the authors to give readers additional information about their work.

eMethods 1. Dietary Intake Assessment

The Oxford WebQ dietary questionnaire was issued at least once to UK Biobank study participants between 2009 and 2012 (n=210,965) on five separate occasions [4]. For this study, participants who completed a minimum of ≥ 2 diet recalls were considered eligible. The Oxford WebQ asked about the frequency of consumption of up to 206 types of food and 32 drinks over the past 24-hours [5]. The Oxford WebQ has been validated previously to represent approximate and true dietary intake [6,7].

© 2023 Thompson AS et al. *JAMA Network Open*.

eMethods 2. Covariate Information

Sociodemographic variables

Sociodemographic factors included sex, age at recruitment (<45, 45-49, 50-54, 55-59, 60-64, 65-73) and ethnicity, which involved grouping participants into six categories (Asian, Black, Multiple, White, Other, or unknown/missing); region, based on the recruitment center the participant attended. The ten regions covered by the 22 assessment centres included: London, Wales, North-West England, North-East England, Yorkshire, West Midlands, East Midlands, South-East England, South-West England, and Scotland; education, with qualification options including College or University degree, A Levels/AS levels or equivalent, O levels/ GCSEs or equivalent, CSEs or equivalent, NVQ or HND or HNC or equivalent, Other professional qualifications eg: nursing, teaching, none of the above, prefer not to answer (participants had the option to select more than one of the qualification options). From this, participants were grouped into categories of 'low', 'medium', 'high', 'none of the above' or 'unknown/missing', based on their highest reported level of education (Low: CSEs or equivalent, O levels/GCSEs or equivalent; Medium: A levels/AS levels or equivalent, NVQ or HND or HNC or equivalent; High: College or University degree, other professional qualifications eg: nursing, teaching; missing/prefer not to say/unknown).

© 2023 Thompson AS et al. *JAMA Network Open*.

Physical activity

Participants were asked to answer questions on their total level of physical activity based on overall intensity. Using this information, excess metabolic equivalent tasks (MET)-hours/week were calculated to reflect the energy expenditure of those who were active during work and recreational time. Following the Internal Physical Activity Questionnaire (IPAQ) scoring guidelines, total physical activity (excess METs) was calculated from the sum of walking (2.3 excess METs), moderate activity (3.0 excess METs), and vigorous activity (7.0 excess METs (for at least 10 minutes of continuous exertion). From this, participants were ranked into quintiles based on their total physical activity level (excess METs hr/ week).

Smoking

Participants were asked to report on their smoking status. This involved the participant answering whether they were a "Never", "Previous" or "Current" smoker. Participants who did not answer this question or those who answered with "prefer not to answer" were categorized as unknown/missing.

© 2023 Thompson AS et al. *JAMA Network Open*.

Alcohol consumption

Participants were asked to answer questions on alcohol consumption at recruitment. For those who reported drinking alcohol, they were asked to answer questions on intake frequency, type of beverage consumed and alcohol consumption with meals. For those who reported to be a former drinker, they were asked to provide the reason why they stopped, including the time since they last drank. From this, participants were categorized into groups of total alcohol intake in grams per day (based their weekly (preferable) or monthly consumption depending on data availability)). Intake groups included, <1g, 1-7g, 8-15g, 16+g per day or unknown/missing. Participants were coded as missing if they reported weekly or monthly alcohol consumption as 'prefer not to say' or 'do not know', except for 'other alcohol' consumption where participants were coded as 0g. If grams/day were 'unknown', but alcohol frequency was 'never' or 'special occasions', an intake of <1g/d was assumed.

Energy intake

Total energy intakes (Kilojoules (KJ)/day)) were derived from the 24hr dietary assessments. A mean energy intake value was calculated using data from all five dietary assessment timepoints. Excluding participants with implausible energy intakes (>17573KJ or < 3347KJ for men and > 14644KJ or < 2092KJ/day for women) [1], participants were ranked into quintiles based on their total energy intake (KJ/day).

© 2023 Thompson AS et al. *JAMA Network Open*.

Sex-specific factors

Data on female reproductive factors were collected at baseline via touchscreen questionnaire. Women were asked questions on age at menarche/parity/menopause, menopausal status, use of exogenous hormones and pregnancy history.

Age at menarche

To establish age at menarche, female participants were asked "How old were you when your periods started?". Following this, participants were provided with the option to answer with their age at menarche, or "do not know" or "prefer not to answer". Women were then categorised into age groups: ≤12 years, 13 years old, or ≥14 years old. Those who answered with "do not know" or "prefer not to answer" were categorised as 'unknown'.

Parity and age at first live birth

To categorise women on parity, women were asked to answer the question, "How many children have you given birth to?". If answered with "1+", the participant was prompted to answer the question, "How old were you when you had your first child?". From this, women were categorised into the number of children birthed, alongside their age at first birth or "do not remember" or "prefer not to answer". For my analysis, women were categorised into age groups: ≤25, 25-29.9 or 30+. Those who responded, "do not remember" or "prefer not to answer" were coded as 'unknown'.

© 2023 Thompson AS et al. *JAMA Network Open*.

Menopausal status

Participants were asked to provide information on menopausal status at recruitment. Women were asked to answer the question, “Have you had your menopause?”. Those who responded with “yes” were prompted with the question, “How old were you when your periods stopped?”. Women were also given the option to answer “no”, “not sure – had hysterectomy”, “not sure – other reason”, or “prefer not to answer”. From this, women were classified as ‘pre-menopausal’, ‘postmenopausal’, or ‘unknown’, depending on their response to a number of other sex-specific factors (see coding below).

	**Pre-MP[a]**	**Pre-MP[b]**	**Pre-MP[c]**	**Post-MP[a]**	**Post-MP[b]**	**Post-MP[c]**	**Unknown[a]**	**Unknown[b]**	**Unknown[c]**
**Sex-specific factors**									
Had menopause									
Yes				X					
No	X						X	X	X
Not sure		X	X		X	X			
Age									
<50 years		X	X						
≥55 years					X				
50-54.9 years									X
Had BO/ hysterectomy									
Yes						X			
No		X	X						
Not sure								X	
Ever used HRT									
Yes									
No		X							
Do not know							X		
Menstruating today									
Yes			X						
No									
Do not know									

Abbreviations: Pre-MP, Pre-menopausal; Post-MP, Post-menopausal; BO, Bilateral oophorectomy; HRT, Hormone replacement therapy.
[a]Pre-menopausal, post-menopausal and unknown menopausal status coding criteria (combination of questions/responses, option one)
[b]Pre-menopausal, post-menopausal and unknown menopausal status coding criteria (combination of questions/responses, option two)
[c]Pre-menopausal, post-menopausal and unknown menopausal status coding criteria (combination of questions/responses, option three)

© 2023 Thompson AS et al. *JAMA Network Open*.

Menopause hormone therapy

When asked about the use of exogenous hormones i.e., menopause hormone therapy (MHT) female participants were questioned about their “ever” use of hormone replacement therapy (HRT). If answered “yes”, participants were prompted with the question: “How old were you when you last used HRT?”. Participants were provided with the option to answer the following: the age they were when they last used HRT, “still taking HRT”, “prefer not to answer” or “do not know”. From this, respondents were categorised into ‘yes’, ‘no’, or ‘missing/unknown’.

Oral contraception

At recruitment, women were asked the question, “Have you ever taken the contraceptive pill?”. Participants had the option to answer with “yes”, “no”, “prefer not to answer” or “do not know”. Following this, respondents were coded as ‘yes’, ‘no’, and those who responded with “prefer not to answer” or “do not know” were coded as ‘unknown’.

Anthropometric measurements

At recruitment, height and weight were measured to determine BMI. To calculate BMI, the weight of each participant (kilograms) was divided by their recorded squared standing height (metres). BMI was coded as <18.5, 18.5-24.99, 25-29.99, 30-34.99, ≥35 kg/m^2. When data on height or weight was missing, participants were categorised as ‘unknown/missing’.

© 2023 Thompson AS et al. *JAMA Network Open*.

Medications and vitamin/mineral supplements

Participants were asked to report their regular medication use against a range of common prescriptions, over-the-counter medications, vitamins, and mineral supplements. Participants could answer if they took medication for cholesterol, blood pressure, or diabetes; pain relief, constipation, or heartburn; vitamins and mineral supplements; and other prescription medications. Female participants also had the option to report if they were taking exogenous hormones. Participants were coded as missing if they reported: “prefer not to say”. Those who reported “yes” to taking regular ‘other’ prescription medications were prompted by a trained interviewer to disclose these medication details. When participants reported “none of the above” or “do not know” to taking regular prescription medication, they were again prompted by an interviewer for confirmation. From this, ‘regular’ medication users were categorised as taking “aspirin” medication. Data obtained through the touchscreen questionnaire and verbal nurse-led interview permitted the name and type of medication to be categorised through a treatment/medicine code. Moreover, participants were asked to report their ‘regular’ vitamin and mineral supplement use from a list of commonly consumed vitamins/minerals. From this, respondents were coded as “non-user” and “regular user”. When data on vitamin and mineral supplement use was missing, or participants answered with “prefer not to say”, participants were categorised as ‘unknown/missing’.

Polypharmacy

At baseline, participants were asked to report the number of treatments (medications) taken. The total number of self-reported medications reported at baseline was used to represent polypharmacy amongst UK Biobank participants [2]. Participants were coded as 0, 1-3, 4-6, 7-9, >10. Those who did not answer this question were recorded as ‘missing’.

© 2023 Thompson AS et al. *JAMA Network Open*.

Multimorbidity

Data on self-reported medical conditions, including number and date of diagnosis, was collected via physician-led interview at baseline assessment. Prevalent morbidity was defined as having one of 43 long-term conditions as determined by Barnett et al. (2012). Participants with two or more long term-conditions (LTCs) were considered as having multimorbidity.

Polygenic risk scores

Polygenic risk scores (PRSs) obtained from external GWAS data provided by Genomics PLC for use upon request within UK Biobank were used for this study. Depending on the outcome of interest and data available, PRSs were categorized into tertiles from low to high genetic risk, or 'missing'. Further detail on the PRS Release has been described elsewhere [3].

© 2023 Thompson AS et al. *JAMA Network Open*.

eMethods 3. Outcome Ascertainment

Outcome – Mortality

All-cause, CVD and cancer mortality was defined by date and underlying cause of death using the International Classification of Disease 10th edition (ICD-10). The ICD-10 codes used to define CVD and cancer mortality included: fatal CVD (I00-I25, I27-I88, I95-I99); fatal cancer (C00-C97, excluding non-melanoma skin cancer: C44). Censoring dates for death data were provided by the NHS Information Centre for participants from England and Wales until September 2021, and the NHS Central Register Scotland for Scotland until October 2021 [8]. For mortality analysis, follow-up was censored using the death registry censoring date or death date, whichever occurred first.

Outcome – CVD

Incident CVD was defined as a primary myocardial infarction (MI) or stroke event using the UK Biobank linked Hospital Inpatient and Death Registry data. For incident CVD analysis, follow-up time was censored at date of hospitalisation, death, or end of follow-up, whichever occurred first. Hospital admission data was available up until September 2021 from the Hospital Episode Statistics (HES) for England, until the end of July 2021 for Scottish Morbidity Records (SMR), and until March 2016 for the Patient Episode Database for Wales (PEDW). Using the ICD-10, any CVD was defined as ischemic heart disease (IHD) (I20), MI (I21-I23, I24.1, I25.2), stroke (I60, I61, I63, or I64). Stroke subtypes were defined as ischemic (I63) and haemorrhagic (I61).

© 2023 Thompson AS et al. *JAMA Network Open*.

Outcome – Cancer

Incident cancer was defined as a primary cancer diagnosis. Cancer diagnosis data was provided through record linkage to National Cancer Registries in England, Wales (follow-up data available from the National Health Service (NHS) information centre until February 2020) and Scotland (follow-up data available from the NHS Central Register Scotland until January 2021). Participants contributed to follow-up time in person years from date of recruitment until the date of first cancer registration, death, or end of follow-up, whichever occurred first. Where Cancer Registry censoring dates did not extend past Hospital Inpatient dates (England and Scotland), participants were further followed using HES and SMR data. PEDW data did not extend past the Cancer Registry data for Wales, so only Cancer Registry dates were used for censoring the end of follow-up. Using ICD-10, cancers were defined as, any cancer (C00-C97, excluding non-melanoma skin cancer: C44), breast cancer (C50), colorectal cancer (C18-C20), and prostate cancer (C61). Breast cancer analyses were coded and restricted to postmenopausal breast cancer.

© 2023 Thompson AS et al. *JAMA Network Open*.

Outcome – Fracture

Using the UK Biobank linked hospital inpatient and death Registry data, incident fractures were defined according to ICD-10 as, any fracture (M484, M485, M800, M808-M8093, M8095-M8099, M843, M844, S12, S22, S32, S42, S52, S72, S82, T02, T08, T10, T12), hip fracture (S720, S721, S722) and vertebrae fracture (M484, M485, S320, S327), due to these fracture sites being most attributable to osteoporosis [9]. Fractures of the face, skull, hands, and feet were excluded from this analysis due to their typically traumatic nature, whilst other traumatic fractures could not be excluded due to a lack of ICD-10 information on the cause of trauma [10]. Hospital admission data was available up until September 2021 from the HES for England, until the end of July 2021 for SMR, and up until March 2016 from the PEDW. For incident fracture analysis, follow-up time was censored at the date of hospitalisation or first reported occurrence, death, or end of follow-up, whichever occurred first.

© 2023 Thompson AS et al. *JAMA Network Open*.

eMethods 4. Multivariable Adjustment

Minimally adjusted models were stratified by region (London, North-West England, North-East England, Yorkshire, West Midlands, East Midlands, South-East England, South-West England, Scotland and Wales), and were adjusted for education (CSEs or equivalent, O levels/GCSEs or equivalent; Medium: A levels/AS levels or equivalent, NVQ or HND or HNC or equivalent; High: College or University degree, Other professional qualifications e.g.: nursing, teaching or Missing/Prefer not to say/Unknown) and sex (male and female).

For all outcomes, multivariable Cox regression models were further adjusted for energy intake (KJ/day) and the following baseline covariates: BMI (<18.5 (underweight), ≥18.5-<25 (normal weight), ≥25-<30 (overweight), ≥30-<35 (obese grade I), ≥35 (obese grade II and III), or unknown/missing), smoking status (never, former, current, or unknown/missing), alcohol intake (none drinkers, <1 g/day, 1-7 g/day, 8-15 g/day, 16+ g/day, or unknown/missing), ethnicity (White, Mixed, Asian or Asian British, Black or Black British, other, or unknown/missing), physical activity (quintiles from low to high metabolic equivalent of task-hours /week, or missing), multimorbidity (number of pre-existing long term-conditions; 0, 1, 2, or >3), polypharmacy (total number of self-reported medications taken at baseline; 0, 1-3, 4-6, 7-9, >10, or missing)

Multivariate models for total mortality were further adjusted for prevalent CVD and cancer (no, yes). CVD-mortality analyses were further adjusted for prevalent CVD, whereas cancer mortality analyses were adjusted for prevalent cancer.

Cox regression analyses on total cancer risk were further adjusted for female-specific factors: menopausal hormone therapy use (no, yes, or unknown/missing among women; or male) and menopausal status at recruitment (premenopausal, postmenopausal, or unknown/missing among women; or male). Analyses on postmenopausal breast cancer risk were further adjusted for age at menarche (≤12, 13, ≥14 years old, or unknown/missing), ever use of oral contraception (no, yes, or unknown/missing), age at first live birth (<25, 25-29.9, ≥30 years of age, or unknown/missing) and polygenic risk score (PRS) for breast cancer (tertiles from low to high PRS for breast cancer or missing), as provided by the UK Biobank.[3] For colorectal cancer analyses, models were further adjusted bowel cancer PRS (tertiles from low to high PRS for bowel

© 2023 Thompson AS et al. *JAMA Network Open*.

cancer or missing). Analyses on prostate cancer analyses were further adjusted for prostate cancer PRS (tertiles from low to high PRS for prostate cancer or missing), but not for female-specific factors.[3]

For total CVD, MI and stroke analyses, multivariable models were further adjusted for a CVD PRS, ischaemic stroke (IS) PRS, or coronary artery disease (CAD) PRS (tertiles from low to high PRS for CVD/ ISS/CAD or missing).[3]

Finally, multivariable Cox regression analyses on fracture were further adjusted for an osteoporosis (OP) PRS (tertiles from low to high PRS for OP, or missing)[3] and vitamin and mineral supplement use (no use, regular use, unknown/missing).

© 2023 Thompson AS et al. *JAMA Network Open*.

eMethods 5. Missing Values

Missing values were treated by using 'missing/unknown' categories in multivariable statistical analyses. The frequencies of missing values were as follows:

- Education level: 6.81%
- BMI: 0.22%
- Smoking status: 0.22%
- Alcohol consumption: 16.73%
- Ethnicity: 0.34%
- Physical activity: 1.82%
- Polypharmacy: 0.02%
- Aspirin use: 27.78%
- Menopausal hormone therapy: 0.23%
- Menopausal status at recruitment: 14.76%
- Age at menarche: 2.58%
- Ever use of oral contraception: 0.17%
- Age at first live birth: 0.04%
- PRS: 2.13% (colon cancer), 1.82% (prostate cancer), 2.18% (CVD, CAD, and IS), and 2.15% (osteoporosis):
- Vitamin and mineral supplement use (0.23%)

© 2023 Thompson AS et al. *JAMA Network Open*.

eTable 1. Comparison of Baseline Characteristics of the UK Biobank Cohort (n = 502 411), Included Participants (n = 126 394), and Excluded Participants (n = 291 446)

	Participants, No. (%)[a]			
	**Whole cohort (N=502,411)**	**Dietary data (≥2 diet recalls) (N=126,394)**	**No dietary data (N=291,446)**	**P-value**
**Characteristics**				<0.001
**Sex-Female**	273,325 (54.4)	70,618 (55.9)	157,114 (53.9)	
**Age at recruitment – years, mean (SD)**	56.5 (8.1)	56.1 (7.8)	56.9 (8.2)	
**BMI- kg/m^2, mean (SD)**	27.4 (4.8)	26.7 (4.6)	27.8 (4.9)	
**Ethnicity**				
Asian	19,131 (3.8)	5,850 (4.6)	9,764 (3.4)	
Black	2,872 (0.6)	466 (0.4)	1,853 (0.6)	
Multiple	18,903 (3.8)	3,535 (2.8)	12,208 (4.2)	
White	454,170 (90.4)	115,371 (91.3)	262,599 (90.1)	
Other[b]	4,557 (0.9)	744 (0.6)	3,016 (1.0)	
**Education**				
Low	125,757 (25.0)	32,365 (25.6)	70,861 (24.3)	
Medium	85,122 (16.9)	20,249 (16.0)	49,628 (17.0)	
High	196,131 (39.0)	65,163 (51.6)	94,692 (32.5)	
Missing	95,391 (19.0)	8,617 (6.8)	76,265 (26.2)	
**Smoking status**				
Never	273,475 (54.4)	72,174 (57.1)	154,482 (53.0)	
Previous	173,024 (34.4)	45,209 (35.8)	98,164 (33.7)	
Current	52,962 (10.5)	8,738 (6.9)	36,416 (12.5)	
Missing	2,950 (0.6)	273 (0.2)	2,384 (0.8)	
**Alcohol intake g/day, mean (SD)**	12.1 (20.8)	12.7 (18.1)	11.7 (22.2)	

Abbreviations: Q, quartile; BMI, body mass index; SD, standard deviation.
[a]Relative frequencies (%) include missing values which may not equate to 100%.
[b]Other includes any race or ethnic group not otherwise specified.

© 2023 Thompson AS et al. *JAMA Network Open*.

eTable 2. Cases, Follow-up Years, and Number Lost to Follow-up for All-Cause Mortality and Incident Cancer, Cardiovascular Disease, and Fracture Outcomes

	Participants, No. (%)			
**Characteristics**	**All-Cause Mortality**	**Any Cancer**	**Any CVD**	**Any Fracture**
**Number of participants**	126,217	117,569	123,134	112,208
**Cases**	5,627 (4.5)	8,939 (7.6)	6,890 (5.6)	4,751 (4.3)
**Follow-up – years, mean (SD)**	12.2 (1.3)	10.6 (1.6)	11.9 (1.9)	11.9 (1.7)
**Person-years**	1,545,827	1,241,046	1,459,597	1,333,865
**Lost to follow-up**	363 (0.3)	349 (0.3)	360 (0.3)	329 (0.3)

Abbreviations: CVD, cardiovascular disease.

© 2023 Thompson AS et al. *JAMA Network Open*.

eTable 3. Key Nutrient Intakes Across Healthful Plant-based Diet Index Quartiles (n = 126 217)

**Key nutrient intakes**	**Mean (SD)**			
**Quartiles of hPDI**	**Q1**	**Q2**	**Q3**	**Q4**
**Participants, No. (%)**	33,901 (26.9)	30,427 (24.1)	30,007 (23.8)	31,882 (25.3)
**hPDI score**	47.7 (3)	53.7 (2)	57.6 (2)	63.4 (3)
**Energy intake kJ/day,**	9099.5 (1825.5)	8518.3 (1798.3)	8226.1 (1796.4)	7984.0 (1811.5)
**Calcium intake mg/day**	1010.2 (304.2)	971.9 (293.2)	965.2 (291.2)	969.2 (294.4)
**Fibre intake g/day**	15.6 (4.9)	16.9 (5.2)	18.2 (5.5)	21.0 (6.3)
**Iodine intake μg/day**	220.9 (88.3)	212.4 (83.0)	207.4 (81.4)	197.2 (81.2)
**Saturated fat intake g/day**	32.2 (10.9)	27.8 (9.8)	25.5 (9.4)	22.6 (8.9)
**Vitamin B12 intake μg/day**	6.6 (2.9)	6.3 (2.7)	6.1 (2.7)	5.7 (2.7)
**Vitamin D intake μg/day**	4.0 (2.4)	3.7 (2.3)	3.6 (2.4)	3.3 (2.4)

Abbreviations: SD, standard deviation; Q, quartile; hPDI, healthful plant-based diet index.

© 2023 Thompson AS et al. *JAMA Network Open*.

eTable 4. Food Groups and Key Nutrient Intakes Across Unhealthful Plant-based Diet Index Quartiles (n = 126 217)

**Key Nutrient Intakes**	**Mean (SD)**			
**Quartiles of uPDI**	**Q1**	**Q2**	**Q3**	**Q4**
**Participants, No. (%)**	34,808 (27.6)	31,273 (24.8)	30,401 (24.1)	29,735 (23.6)
**uPDI score**	46.6 (3.2)	52.4 (1.5)	56.3 (1.5)	61.8 (2.9)
**Energy intake kJ/day**	8854.7 (1867.3)	8500.9 (1832.7)	8319.3 (1820.2)	8141.1 (1828.6)
**Calcium intake mg/day**	1086.0 (306.1)	1001.2 (287.0)	943.6 (275.4)	870.4 (269.3)
**Fibre intake g/day**	21.2 (6.1)	18.5 (5.3)	16.8 (4.9)	14.5 (4.6)
**Iodine intake µg/day**	238.3 (90.0)	215.3 (81.7)	201.1 (77.9)	179.0 (72.8)
**Saturated fat intake g/day**	28.8 (11.2)	27.2 (10.4)	26.5 (9.9)	25.7 (9.6)
**Vitamin B12 intake µg/day**	7.2 (3.0)	6.3 (2.7)	5.8 (2.5)	5.2 (2.3)
**Vitamin D intake µg/day**	4.4 (2.8)	3.7 (2.3)	3.4 (2.1)	3.0 (1.8)

Abbreviations: SD, standard deviation; Q, quartile; uPDI, unhealthful plant-based diet index.

© 2023 Thompson AS et al. *JAMA Network Open*.

eTable 5. Baseline Characteristics Across Unhealthful Plant-based Diet Index Quartiles (n = 126 217)

Characteristics across uPDI	Participants, No. (%)[a]			
	Q1	Q2	Q3	Q4
**Number of participants**	34,808 (27.6)	31,273 (24.8)	30,401 (24.1)	29,735 (23.6)
**uPDI, mean (SD)**	46.6 (3.2)	52.4 (1.5)	56.3 (1.5)	61.8 (2.9)
**Sex-Female**	18,318 (52.6)	17,482 (55.9)	17,210 (56.6)	17,545 (59.0)
**Age at recruitment – years, mean (SD)**	57.5 (7.4)	56.7 (7.6)	56.0 (7.8)	54.1 (8.1)
**BMI- kg/m^2, mean (SD)**	26.5 (4.4)	26.5 (4.5)	26.7 (4.5)	27.2 (4.9)
**Physical activity**				
Low	9,735 (28.0)	9,797 (31.3)	10,379 (34.1)	11,415 (38.4)
Moderate	11,570 (33.2)	10,575 (33.8)	9,948 (32.7)	9,205 (31.0)
High	12,986 (37.3)	10,314 (33.0)	9,523 (31.3)	8,469 (28.5)
**Ethnicity**				
Asian	1,990 (5.7)	1,440 (4.6)	1,323 (4.4)	1,091 (3.7)
Black	127 (0.4)	107 (0.3)	105 (0.4)	127 (0.4)
Multiple	930 (2.7)	826 (2.6)	879 (2.9)	895 (3.0)
White	31,432 (90.3)	28,610 (91.4)	27,806 (91.5)	27,360 (92.0)
Other[b]	195 (0.6)	187 (0.6)	186 (0.6)	175 (0.6)
**Education**				
Low	8,162 (23.5)	7,742 (24.8)	7,917 (26.0)	8,505 (28.6)
Medium	5,045 (14.5)	4,764 (15.2)	4,975 (16.4)	5,437 (18.3)
High	19,614 (56.4)	16,782 (53.7)	15,275 (50.3)	13,408 (45.1)
**Smoking status**				
Never	18,832 (54.1)	17,768 (56.8)	17,550 (57.7)	17,953 (60.4)
Previous	13,776 (40.0)	11,445 (36.6)	10,661 (35.1)	9,230 (31.0)
Current	2,134 (6.1)	1,982 (6.3)	2,115 (7.0)	2,488 (8.4)
**Alcohol intake g/day, mean (SD)**	13.5 (18.5)	12.8 (17.5)	12.5 (17.9)	11.6 (18.5)
**Medication use**				
Aspirin	4,992 (14.3)	4,054 (13.0)	3,944 (13.0)	3,592 (12.1)
**Multimorbidity**				
0 LTCs	14,210 (40.8)	13,037 (41.7)	12,758 (42.0)	12,369 (41.6)
1 LTC	10,602 (30.5)	9,417 (30.1)	9,103 (29.9)	8,979 (30.2)
2 LTCs	5,830 (16.8)	5,183 (16.6)	5,039 (16.6)	4,780 (16.1)
≥3 LTCs	4,166 (12.0)	3,636 (11.6)	3,501 (11.5)	3,607 (12.1)
**Polypharmacy**				
0	10,965 (31.5)	9,834 (31.4)	9,449 (31.1)	8,936 (30.1)
1-3	16,047 (46.1)	14,532 (46.5)	14,186 (46.7)	14,086 (47.4)
4-6	5,755 (16.5)	5,128 (16.4)	5,015 (16.5)	4,871 (16.4)
7-9	1,536 (4.4)	1,334 (4.3)	1,269 (4.2)	1,312 (4.4)
≥10	499 (1.4)	451 (1.4)	477 (1.6)	525 (1.8)
**Unhealthful Plant-Based Diet Index food item intake, portions/day[c]**				
Wholegrains[d]	2.9 (1.5)	2.3 (1.4)	1.9 (1.3)	1.4 (1.1)
Fruit[d]	3.0 (1.7)	2.4 (1.5)	2.0 (1.4)	1.4 (1.2)
Vegetables[d]	3.4 (2.1)	2.6 (1.8)	2.1 (1.6)	1.6 (1.3)
Nuts[d]	0.3 (0.5)	0.2 (0.3)	0.1 (0.3)	0.1 (0.2)
Legumes[d]	0.5 (0.5)	0.4 (0.4)	0.3 (0.4)	0.3 (0.4)
Tea and coffee[d]	4.9 (1.6)	4.5 (1.6)	4.2 (1.6)	3.7 (1.6)
Refined grains[e]	0.7 (0.8)	1.0 (1.0)	1.2 (1.1)	1.5 (1.1)
Potatoes[e]	0.6 (0.5)	0.7 (0.5)	0.7 (0.5)	0.8 (0.6)
Sugary drinks[e]	0.3 (0.6)	0.4 (0.7)	0.5 (0.8)	0.9 (1.0)
Fruit juices[e]	0.4 (0.5)	0.4 (0.5)	0.5 (0.5)	0.6 (0.6)
Sweets and desserts[e]	1.1 (1.0)	1.3 (1.1)	1.5 (1.2)	1.8 (1.3)
Animal fat	0.9 (1.1)	0.7 (1.0)	0.6 (0.9)	0.5 (0.8)

© 2023 Thompson AS et al. *JAMA Network Open*.

Dairy	1.3 (0.8)	1.1 (0.7)	1.0 (0.7)	0.8 (0.7)
Eggs	0.4 (0.5)	0.3 (0.4)	0.2 (0.4)	0.2 (0.3)
Fish or seafood	0.5 (0.4)	0.3 (0.4)	0.3 (0.4)	0.2 (0.3)
Meat	1.2 (0.9)	1.2 (0.8)	1.0 (0.8)	1.1 (0.8)
Miscellaneous animal-based foods	0.1 (0.3)	0.1 (0.3)	0.1 (0.3)	0.1 (0.3)

Abbreviations: Q, quartile; uPDI, unhealthful plant-based diet index; BMI, body mass index; SD, standard deviation; LTC, long-term condition.

[a]Relative frequencies (%) include missing values which may not equate to 100%.

[b]Other includes any race or ethnic group not otherwise specified.

[c]Portions sizes were specified as a "serving" in the Oxford WebQ.

[d]Healthy plant-foods

[e]Unhealthy plant-foods

© 2023 Thompson AS et al. *JAMA Network Open*.

eTable 6. Intraclass Correlation Coefficients for Reproducibility of Plant-based Diet Index Scores Over Time

24-hr dietary recall cycles[a]	Ethnic Group					
**T2+T3 *vs.* T4+T5**	**Overall**	**Asian**	**Black**	**Multiple**	**White**	**Other[b]**
**Number of participants**	24,893	1,116	72	554	22,945	122
**hPDI**	0.58	0.65	0.54	0.62	0.58	0.62
**uPDI**	0.55	0.54	0.44	0.60	0.55	0.43

Abbreviations: hPDI, healthful plant-based diet index; uPDI, unhealthful plant-based diet index

[a]Intra-class correlation coefficients are calculated from the means of cycles 2 and 3, correlated with the means of cycles 4 and 5.

[b]Other includes any race or ethnic group not otherwise specified.

© 2023 Thompson AS et al. *JAMA Network Open*.

eTable 7. Hazard Ratios (95% CIs) of Mortality Across Sex-Specific Healthful Plant-based Diet Index Quartiles (n = 126 217)

**hPDI**	Q1	Q2	Q3	Q4	P-trend	P-corrected
**Outcome**						
**All-Cause Mortality**						
Cases/total	1,602/33,901	1,375/30,427	1,269/30,007	1,381/31,882		
HR (95% CI)[a]	1.00[c]	0.84 (0.78-0.90)	0.75 (0.70-0.81)	0.76 (0.70- 0.81)	<0.001	0.004
HR (95% CI)[b]	1.00[c]	0.90 (0.83-0.97)	0.82 (0.76-0.89)	0.84 (0.78-0.91)	<0.001	0.004
**Cancer-Mortality**						
Cases/total	873/33,901	803/30,427	758/30,007	841/31,882		
HR (95% CI)[a]	1.00[c]	0.90 (0.82-1.00)	0.83 (0.75-0.92)	0.85 (0.77- 0.93)	<0.001	0.004
HR (95% CI)[b]	1.00[c]	0.96 (0.87-1.06)	0.91 (0.82-1.00)	0.95 (0.86- 1.05)	0.10	0.22
**CVD-Mortality**						
Cases/total	195/33,901	178/30,427	158/30,007	167/31,882		
HR (95% CI)[a]	1.00[c]	0.89 (0.73-1.09)	0.77 (0.62-0.94)	0.75 (0.61- 0.92)	0.001	0.004
HR (95% CI)[b]	1.00[c]	0.97 (0.79-1.19)	0.86 (0.69-1.06)	0.85 (0.69- 1.06)	0.07	0.16

Abbreviations: Q, quartile; hPDI, healthful plant-based diet index; CVD, Cardiovascular Disease; HR, hazard ratios; CI, confidence intervals.

P-trend is for linear trend.

P-corrected is the P-trend corrected for multiple testing.

[a]Hazard Ratios with 95% Confidence Intervals (CI), adjusted for sex and education; stratified by region.

[b]Hazard Ratios with 95% Confidence Intervals (CI), adjusted for sex, BMI, ethnicity, physical activity, smoking status, alcohol intake, education, energy intake, polypharmacy index, multimorbidity index and aspirin use; stratified by region.

For all-cause mortality analyses, models were further adjusted for prevalent CVD and prevalent cancer.

For CVD mortality analyses, models were further adjusted for prevalent CVD.

For cancer mortality analyses, models were further adjusted for prevalent cancer.

[c]Reference categories

© 2023 Thompson AS et al. *JAMA Network Open*.

eTable 8. Hazard Ratios (95% CIs) of Mortality Across Sex-Specific Unhealthful Plant-based Diet Index Quartiles (n = 126 217)

uPDI	Q1	Q2	Q3	Q4	P-trend	P-corrected
**Outcome**						
**All-Cause Mortality**						
Cases/total	1,579/34,808	1,399/31,273	1,352/30,401	1,297/29,735		
HR (95% CI)[a]	1.00[c]	1.06 (0.99-1.14)	1.12 (1.04-1.21)	1.30 (1.21- 1.40)	<0.001	0.004
HR (95% CI)[b]	1.00[c]	1.06 (0.99-1.14)	1.09 (1.02-1.18)	1.23 (1.14-1.32)	<0.001	0.004
**Cancer-Mortality**						
Cases/total	917/34,808	813/31,273	809/30,401	736/29,735		
HR (95% CI)[a]	1.00[c]	1.05 (0.96-1.16)	1.14 (1.04-1.25)	1.24 (1.13- 1.37)	<0.001	0.004
HR (95% CI)[b]	1.00[c]	1.06 (0.96-1.16)	1.12 (1.02-1.23)	1.19 (1.08- 1.32)	<0.001	0.004
**CVD-Mortality**						
Cases/total	205/34,808	184/31,273	164/30,401	145/29,735		
HR (95% CI)[a]	1.00[c]	1.09 (0.89-1.33)	1.06 (0.86-1.30)	1.16 (0.94- 1.43)	0.04	0.08
HR (95% CI)[b]	1.00[c]	1.09 (0.89-1.33)	1.04 (0.84-1.28)	1.08 (0.87- 1.34)	0.15	0.25

Abbreviations: Q, quartile; uPDI, unhealthful plant-based diet index; CVD, cardiovascular disease; HR, hazard ratios; CI, confidence intervals.

P-trend is for linear trend.

P-corrected is the P-trend corrected for multiple testing.

[a]Hazard Ratios with 95% Confidence Intervals (CI), adjusted for sex and education; stratified by region.

[b]Hazard Ratios with 95% Confidence Intervals (CI), adjusted for sex, BMI, ethnicity, physical activity, smoking status, alcohol intake, education, energy intake, polypharmacy index, multimorbidity index and aspirin use; stratified by region.

For all-cause mortality analyses, models were further adjusted for prevalent CVD and prevalent cancer.

For CVD mortality analyses, models were further adjusted for prevalent CVD.

For cancer mortality analyses, models were further adjusted for prevalent cancer.

[c]Reference categories

© 2023 Thompson AS et al. *JAMA Network Open*.

eTable 9. Hazard Ratios (95% CIs) of Cancer (Postmenopausal Breast, Colorectal, Prostate, or Any) Across Sex-Specific Healthful Plant-based Diet Index Quartiles (n = 117 569)

hPDI	Q1	Q2	Q3	Q4	P-trend	P-corrected
**Outcome**						
**Any Cancer**						
Cases/total	2,331/31,642	2,178/28,348	2,162/27,930	2,268/29,649		
HR (95% CI)[a]	1.00[c]	0.94 (0.89-1.00)	0.91 (0.85-0.96)	0.87 (0.82- 0.92)	<0.001	0.004
HR (95% CI)[b]	1.00[c]	0.97 (0.91-1.03)	0.95 (0.90-1.01)	0.93 (0.88-0.99)	0.01	0.03
**Postmenopausal Breast Cancer**						
Cases/total	228/16,759	276/15,790	288/15,849	291/16,834		
HR (95% CI)[a]	1.00[c]	1.07 (0.90-1.27)	1.05 (0.88-1.24)	0.95 (0.80- 1.13)	0.21	0.29
HR (95% CI)[b]	1.00[c]	1.07 (0.89-1.28)	1.07 (0.89-1.28)	0.99 (0.83- 1.19)	0.52	0.61
**Colorectal Cancer**						
Cases/total	260/31,642	249/28,348	227/27,930	223/29,649		
HR (95% CI)[a]	1.00[c]	0.96 (0.81-1.15)	0.85 (0.71-1.02)	0.77 (0.64- 0.92)	0.002	0.007
HR (95% CI)[b]	1.00[c]	1.01 (0.85-1.21)	0.92 (0.77-1.11)	0.87 (0.72- 1.05)	0.11	0.22
**Prostate Cancer**						
Cases/total	515/14,883	514/12,558	540/12,081	568/12,815		
HR (95% CI)[a]	1.00[c]	1.01 (0.90-1.15)	1.03 (0.91-1.16)	0.98 (0.87- 1.10)	0.49	0.61
HR (95% CI)[b]	1.00[c]	1.00 (0.89-1.14)	1.02 (0.90-1.16)	0.98 (0.86- 1.11)	0.50	0.61

Abbreviations: Q, quartile; hPDI, healthful plant-based diet index; BC, breast cancer; PRS, polygenic risk score; BMI, body mass index; CRC, colorectal cancer; MHT, menopause hormone therapy; PC, prostate Cancer; HR, hazard ratios; CI, confidence intervals.

P-trend is for linear tren.

P-corrected is the P-trend corrected for multiple testing.

[a]Hazard Ratios with 95% Confidence Intervals (CI), adjusted for sex and education; stratified by region.

[b]Hazard Ratios with 95% Confidence Intervals (CI), adjusted for sex (excluding breast and prostate cancer analyses), BMI, ethnicity, physical activity, smoking status, alcohol intake, education, energy intake, polypharmacy index, multimorbidity index and aspirin use; stratified by region.

For any cancer analyses, models were further adjusted for menopausal status and use of MHT.

For breast cancer analyses, models were restricted to postmenopausal breast cancer cases and were further adjusted for use of MHT, use of oral contraception, PRS (BC), age at menarche and age at first live birth.

For colorectal cancer analyses, models were further adjusted for menopausal status, PRS (CRC) and MHT.

For prostate cancer analyses, models were further adjusted for PRS (PC).

[c]Reference categories.

© 2023 Thompson AS et al. *JAMA Network Open*.

eTable 10. Hazard Ratios (95% CIs) of Cancer (Postmenopausal Breast, Colorectal, Prostate, or Any) Across Sex-Specific Unhealthful Plant-based Diet Index Quartiles (n = 117 569)

**uPDI**	Q1	Q2	Q3	Q4	P-trend	P-corrected
**Outcome**						
**Any Cancer**						
Cases/total	2,622/33,372	2,260/29,079	2,117/28,302	1,940/27,816		
HR (95% CI)[a]	1.00[c]	1.02 (0.97-1.08)	1.04 (0.98-1.10)	1.12 (1.06- 1.19)	<0.001	0.004
HR (95% CI)[b]	1.00[c]	1.03 (0.97-1.09)	1.04 (0.98-1.10)	1.10 (1.03-1.17)	<0.001	0.004
**Postmenopausal Breast Cancer**						
Cases/total	306/16,855	288/16,170	258/15,925	231/16,282		
HR (95% CI)[a]	1.00[c]	1.06 (0.90-1.25)	1.06 (0.90-1.25)	1.17 (0.98- 1.39)	0.11	0.22
HR (95% CI)[b]	1.00[c]	1.07 (0.91-1.26)	1.07 (0.90-1.26)	1.16 (0.97- 1.38)	0.13	0.23
**Colorectal Cancer**						
Cases/total	268/33,372	265/29,079	244/28,302	182/27,816		
HR (95% CI)[a]	1.00[c]	1.19 (1.00-1.40)	1.18 (0.99-1.41)	1.04 (0.86- 1.26)	0.14	0.24
HR (95% CI)[b]	1.00[c]	1.20 (1.01-1.42)	1.19 (1.00-1.42)	1.05 (0.86- 1.27)	0.12	0.22
**Prostate Cancer**						
Cases/total	717/15,517	540/12,909	482/12,377	398/11,534		
HR (95% CI)[a]	1.00[c]	0.97 (0.87-1.09)	0.97 (0.86-1.09)	1.02 (0.90- 1.15)	0.73	0.80
HR (95% CI)[b]	1.00[c]	0.99 (0.88-1.11)	0.99 (0.88-1.11)	1.05 (0.93- 1.19)	0.45	0.57

Abbreviations: Q, quartile; uPDI, unhealthful plant-based diet index; BC, breast cancer; PRS, polygenic risk score; BMI, body mass index; CRC, colorectal cancer; MHT, menopause hormone therapy; PC, prostate Cancer; HR, hazard ratios; CI, confidence intervals

P-trend is for linear trend.

P-corrected is the P-trend corrected for multiple testing..

[a]Hazard Ratios with 95% Confidence Intervals (CI), adjusted for sex and education; stratified by region.

[b]Hazard Ratios with 95% Confidence Intervals (CI), adjusted for sex (excluding breast and prostate cancer analyses), BMI, ethnicity, physical activity, smoking status, alcohol intake, education, energy intake, polypharmacy index, multimorbidity index and aspirin use; stratified by region.

For any cancer analyses, models were further adjusted for menopausal status and use of MHT.

For breast cancer analyses, models were restricted to postmenopausal breast cancer cases and were further adjusted for use of MHT, use of oral contraception, PRS (BC), age at menarche and age at first live birth.

For colorectal cancer analyses, models were further adjusted for menopausal status, PRS (CRC) and MHT.

For prostate cancer analyses, models were further adjusted for PRS (PC).

[c]Reference categories.

© 2023 Thompson AS et al. *JAMA Network Open*.

eTable 11. Hazard Ratios (95% CIs) of Cardiovascular Disease (Myocardial Infarction, Stroke, or Any) Across Sex-Specific Healthful Plant-based Diet Index Quartiles (n = 123 134)

**hPDI**	Q1	Q2	Q3	Q4	P-trend	P-corrected
**Outcome**						
**Any CVD**						
Cases/total	1,839/33,108	1,679/29,656	1,623/29,264	1,749/31,106		
HR (95% CI)[a]	1.00[c]	0.92 (0.86-0.98)	0.87 (0.81-0.93)	0.86 (0.80- 0.92)	<0.001	0.004
HR (95% CI)[b]	1.00[c]	0.95 (0.89-1.02)	0.92 (0.86-0.99)	0.92 (0.86-0.99)	0.002	0.007
**Ischaemic stroke**						
Cases/total	307/33,108	295/29,656	265/29,264	284/31,106		
HR (95% CI)[a]	1.00[c]	0.94 (0.80-1.10)	0.81 (0.69-0.95)	0.79 (0.68- 0.93)	0.002	0.007
HR (95% CI)[b]	1.00[c]	0.96 (0.82-1.13)	0.85 (0.72-1.00)	0.84 (0.71- 0.99)	0.03	0.08
**Haemorrhagic stroke**						
Cases/total	127/33,108	133/29,656	101/29,264	128/31,106		
HR (95% CI)[a]	1.00[c]	0.85 (0.66-1.10)	0.74 (0.57-0.96)	0.85 (0.66- 1.08)	0.06	0.15
HR (95% CI)[b]	1.00[c]	0.90 (0.69-1.16)	0.80 (0.61-1.05)	0.92 (0.71- 1.20)	0.30	0.44
**MI**						
Cases/total	899/33,108	758/29,656	797/29,264	799/31,106		
HR (95% CI)[a]	1.00[c]	0.85 (0.77-0.94)	0.89 (0.81-0.98)	0.81 (0.74- 0.89)	<0.001	0.004
HR (95% CI)[b]	1.00[c]	0.88 (0.80-0.97)	0.94 (0.85-1.04)	0.86 (0.78- 0.95)	<0.001	0.004

Abbreviations: Q, quartile; hPDI, healthful plant-based diet index; BMI, Body Mass Index; CVD, cardiovascular disease; PRS, polygenic risk score; IS, Ischaemic stroke; MI, myocardial infarction; CAD, coronary artery disease; HR, hazard ratios; CI, confidence intervals.

P-trend is for linear trend.

P-corrected is the P-trend corrected for multiple testing.

[a]Hazard Ratios with 95% Confidence Intervals (CI), adjusted for sex and education; stratified by region.

[b]Hazard Ratios with 95% Confidence Intervals (CI), adjusted for sex, BMI, ethnicity, physical activity, smoking status, alcohol intake, education, energy intake, polypharmacy index, multimorbidity index and aspirin use; stratified by region.

For any CVD and haemorrhagic stroke, models were further adjusted for PRS (CVD).

For ischaemic stroke analyses, models were further adjusted for PRS (IS).

For MI, models were further adjusted for PRS (CAD).

[c]Reference categories.

© 2023 Thompson AS et al. *JAMA Network Open*.

eTable 12. Hazard Ratios (95% CIs) of Cardiovascular Disease (Myocardial Infarction, Stroke, or Any) Across Sex-Specific Unhealthful Plant-based Diet Index Quartiles (n = 123 134)

uPDI	Q1	Q2	Q3	Q4	P-trend	P-corrected
**Outcome**						
**Any CVD**						
Cases/total	2,023/33,970	1,671/30,494	1,699/29,637	1,497/29,033		
HR (95% CI)[a]	1.00[c]	1.00 (0.94-1.07)	1.11 (1.04-1.118)	1.18 (1.10- 1.26)	<0.001	0.004
HR (95% CI)[b]	1.00[c]	1.01 (0.95-1.08)	1.09 (1.03-1.17)	1.21 (1.05-1.20)	<0.001	0.004
**Ischaemic stroke**						
Cases/total	358/33,970	266/30,494	274/29,637	253/29,033		
HR (95% CI)[a]	1.00[c]	0.90 (0.77-1.06)	1.03 (0.88-1.20)	1.17 (1.00- 1.38)	<0.001	0.004
HR (95% CI)[b]	1.00[c]	0.90 (0.77-1.06)	1.01 (0.87-1.19)	1.23 (0.95- 1.33)	0.008	0.02
**Haemorrhagic stroke**						
Cases/total	144/33,970	124/30,494	106/29,637	95/29,033		
HR (95% CI)[a]	1.00[c]	1.07 (0.83-1.35)	1.00 (0.78-1.29)	1.13 (0.87- 1.46)	0.15	0.25
HR (95% CI)[b]	1.00[c]	1.06 (0.83-1.36)	0.98 (0.76-1.26)	1.06 (0.82- 1.39)	0.33	0.46
**MI**						
Cases/total	931/33,970	778/30,494	829/29,637	715/29,033		
HR (95% CI)[a]	1.00[c]	1.02 (0.93-1.12)	1.18 (1.08-1.30)	1.23 (1.12- 1.36)	<0.001	0.004
HR (95% CI)[b]	1.00[c]	1.03 (0.94-1.13)	1.17 (1.07-1.29)	1.17 (1.06- 1.29)	<0.001	0.004

Abbreviations: Q, quartile; uPDI, unhealthful plant-based diet index; BMI, Body Mass Index; CVD, cardiovascular disease; PRS, polygenic risk score; IS, Ischaemic stroke; MI, myocardial infarction; CAD, coronary artery disease; HR, hazard ratios; CI, confidence intervals.

P-trend is for linear trend.

P-corrected is the P-trend corrected for multiple testing.

[a]Hazard Ratios with 95% Confidence Intervals (CI), adjusted for sex and education; stratified by region.

[b]Hazard Ratios with 95% Confidence Intervals (CI), adjusted for sex, BMI, ethnicity, physical activity, smoking status, alcohol intake, education, energy intake, polypharmacy index, multimorbidity index and aspirin use; stratified by region.

For any CVD and haemorrhagic stroke, models were further adjusted for PRS (CVD).

For ischaemic stroke analyses, models were further adjusted for PRS (IS).

For MI, models were further adjusted for PRS (CAD).

[c]Reference categories.

© 2023 Thompson AS et al. *JAMA Network Open*.

eTable 13. Hazard Ratios (95% CIs) of Fracture (Hip, Vertebrae, or Any) Across Sex-Specific Healthful Plant-based Diet Index Quartiles (n = 112 208)

**hPDI**	Q1	Q2	Q3	Q4	P-trend	P-corrected
**Outcome**						
**Any Fracture**						
Cases/total	1,151/30,283	1,104/27,008	1,198/26,721	1,298/28,196		
HR (95% CI)[a]	1.00[c]	0.96 (0.89-1.05)	1.01 (0.92-1.10)	1.02 (0.94- 1.10)	0.58	0.68
HR (95% CI)[b]	1.00[c]	0.98 (0.90-1.06)	1.02 (0.94-1.11)	1.03 (0.95-1.12)	0.45	0.57
**Hip Fracture**						
Cases/total	176/30,283	166/27,008	175/26,721	219/28,196		
HR (95% CI)[a]	1.00[c]	0.89 (0.72-1.10)	0.89 (0.72-1.10)	1.03 (0.84- 1.26)	0.69	0.78
HR (95% CI)[b]	1.00[c]	0.91 (0.73-1.13)	0.91 (0.73-1.13)	1.03 (0.84- 1.27)	0.70	0.78
**Vertebrae Fracture**						
Cases/total	85/30,283	71/27,008	73/26,721	90/28,196		
HR (95% CI)[a]	1.00[c]	0.83 (0.61-1.14)	0.83 (0.61-1.13)	0.95 (0.70- 1.28)	0.35	0.47
HR (95% CI)[b]	1.00[c]	0.86 (0.63-1.19)	0.88 (0.64-1.22)	1.05 (0.77- 1.44)	0.80	0.86

Abbreviations: Q, quartile; hPDI, healthful plant-based diet index; PRS, polygenic risk score; OP, Osteoporosis; BMI, Body Mass Index; HR, hazard ratios; CI, confidence intervals.

P-trend is for linear trend.

P-corrected is the P-trend corrected for multiple testing.

[a]Hazard Ratios with 95% Confidence Intervals (CI), adjusted for sex and education; stratified by age, region.

[b]Hazard Ratios with 95% Confidence Intervals (CI), adjusted for sex, BMI, ethnicity, physical activity, smoking status, alcohol intake, education, energy intake, vitamin/mineral supplement use, PRS (OP), polypharmacy index, multimorbidity index and aspirin use; stratified by age, region.

[c]Reference categories.

© 2023 Thompson AS et al. *JAMA Network Open*.

eTable 14. Hazard Ratios (95% CIs) of Fracture (Hip, Vertebrae, or Any) Across Sex-Specific Unhealthful Plant-based Diet Index Quartiles (n = 112 208)

**uPDI**	Q1	Q2	Q3	Q4	P-trend	P-corrected
**Outcome**						
**Any Fracture**						
Cases/total	1,364/30,728	1,204/27,835	1,325/30,187	858/23,458		
HR (95% CI)[a]	1.00[c]	1.00 (0.93-1.09)	1.05 (0.97-1.14)	1.03 (0.94- 1.12)	0.35	0.47
HR (95% CI)[b]	1.00[c]	1.01 (0.93-1.09)	1.06 (0.98-1.14)	1.03 (0.95-1.13)	0.29	0.44
**Hip Fracture**						
Cases/total	217/30,728	212/27,835	179/30,187	128/23,458		
HR (95% CI)[a]	1.00[c]	1.14 (0.95-1.38)	0.95 (0.78-1.16)	1.10 (0.88- 1.38)	0.89	0.91
HR (95% CI)[b]	1.00[c]	1.15 (0.95-1.39)	0.95 (0.78-1.16)	1.12 (0.89- 1.40)	0.95	0.95
**Vertebrae Fracture**						
Cases/total	100/30,728	77/27,835	80/30,187	62/23,458		
HR (95% CI)[a]	1.00[c]	0.90 (0.67-1.21)	0.91 (0.68-1.22)	1.05 (0.76- 1.44)	0.91	0.92
HR (95% CI)[b]	1.00[c]	0.89 (0.66-1.20)	0.89 (0.66-1.20)	1.01 (0.73- 1.40)	0.94	0.95

Abbreviations: Q, quartile; uPDI, unhealthful plant-based diet index; PRS, polygenic risk score; OP, Osteoporosis; BMI, Body Mass Index; HR, hazard ratios; CI, confidence intervals.

P-trend is for linear trend.

P-corrected is the P-trend corrected for multiple testing.

[a]Hazard Ratios with 95% Confidence Intervals (CI), adjusted for sex and education; stratified by region.

[b]Hazard Ratios with 95% Confidence Intervals (CI), adjusted for sex, BMI, ethnicity, physical activity, smoking status, alcohol intake, education, energy intake, vitamin/mineral supplement use, PRS (OP), polypharmacy index, multimorbidity index and aspirin use; stratified by region.

[c]Reference categories.

© 2023 Thompson AS et al. *JAMA Network Open*.

eFigure 1. Flow Diagram of UK Biobank Participants Excluded From Analysis

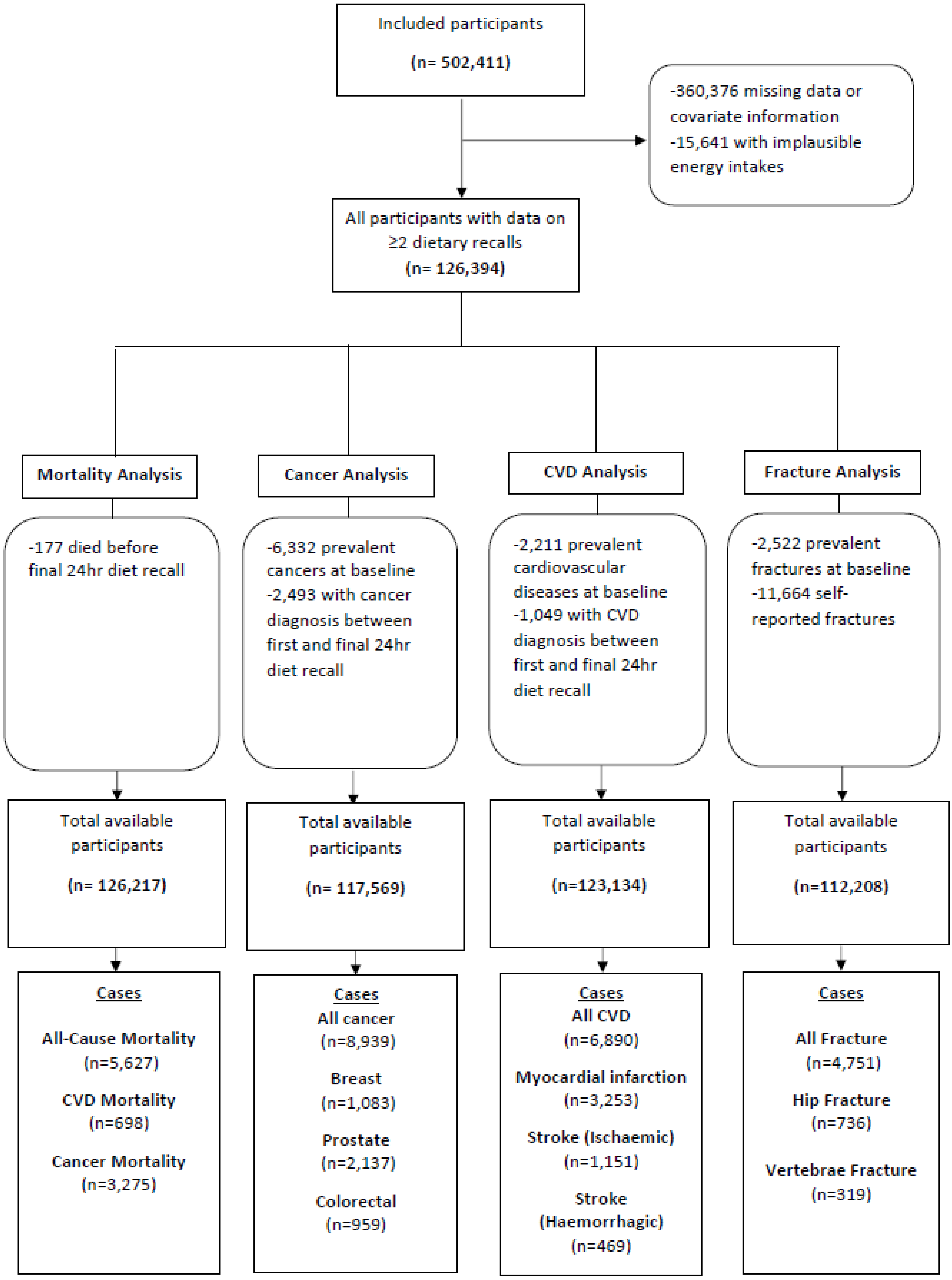

© 2023 Thompson AS et al. *JAMA Network Open*.

eFigure 2. Histogram Showing Total Healthful Plant-based Diet Index (hPDI) Scores of UK Biobank Participants (n = 126 217)

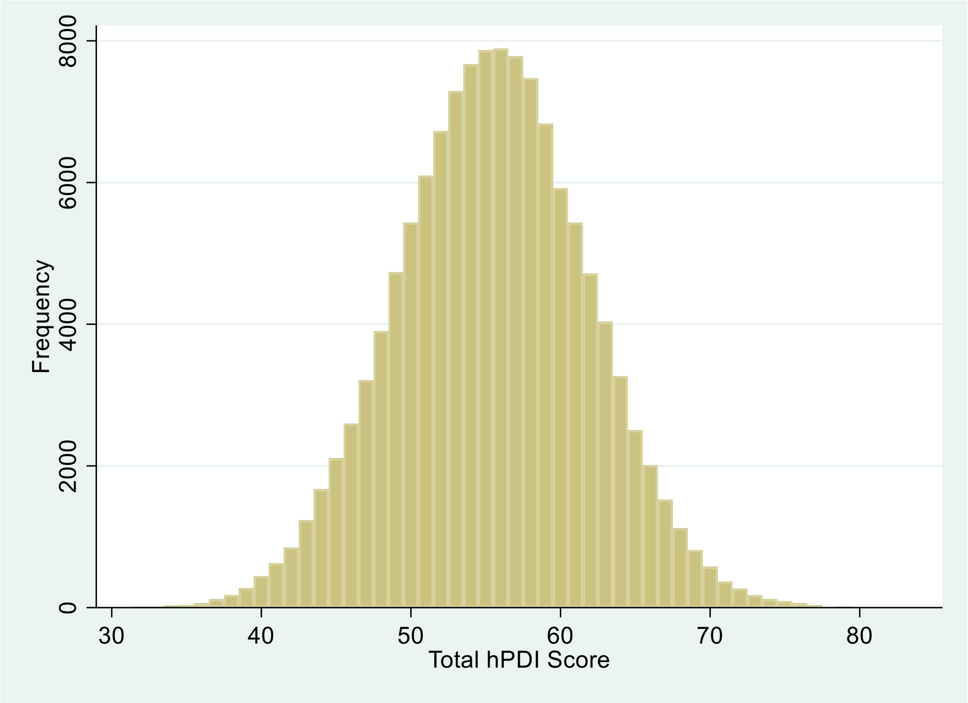

eFigure 3. Histogram Showing Total Unhealthful Plant-based Diet Index (uPDI) Scores of UK Biobank Participants (n = 126 217)

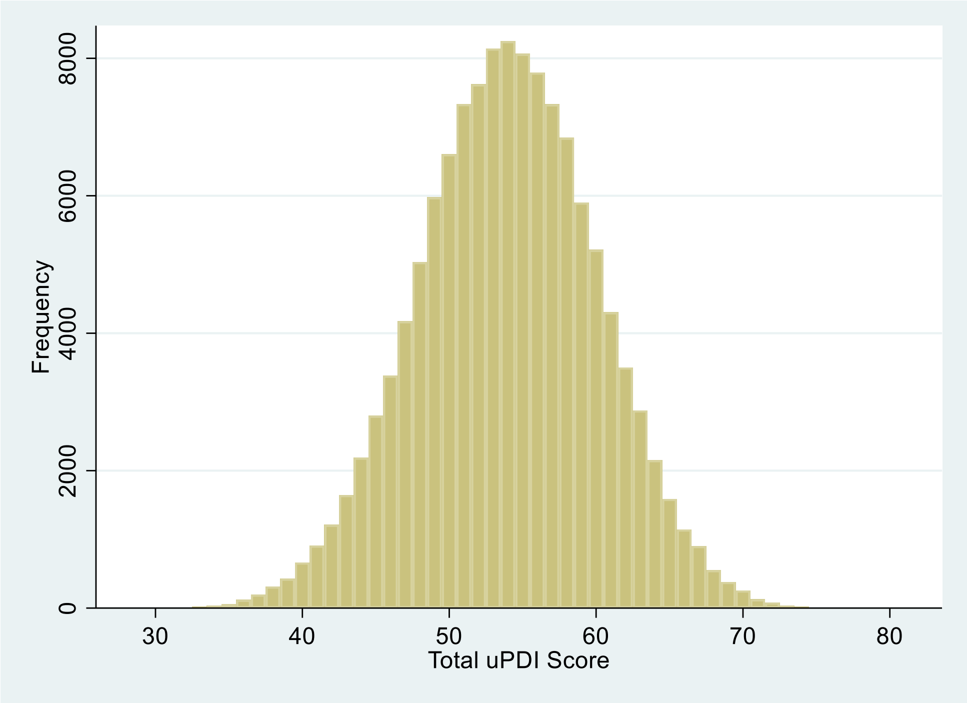

© 2023 Thompson AS et al. *JAMA Network Open*.

eFigure 4. Cubic Spline Graphs Showing Fully Adjusted Linear Associations of Healthful Plant-based Diet Index (hPDI) and Unhealthful Plant-based Diet Index (uPDI) Scores With All-Cause Mortality, Cancer, Cardiovascular Disease (CVD), and Fracture Risk

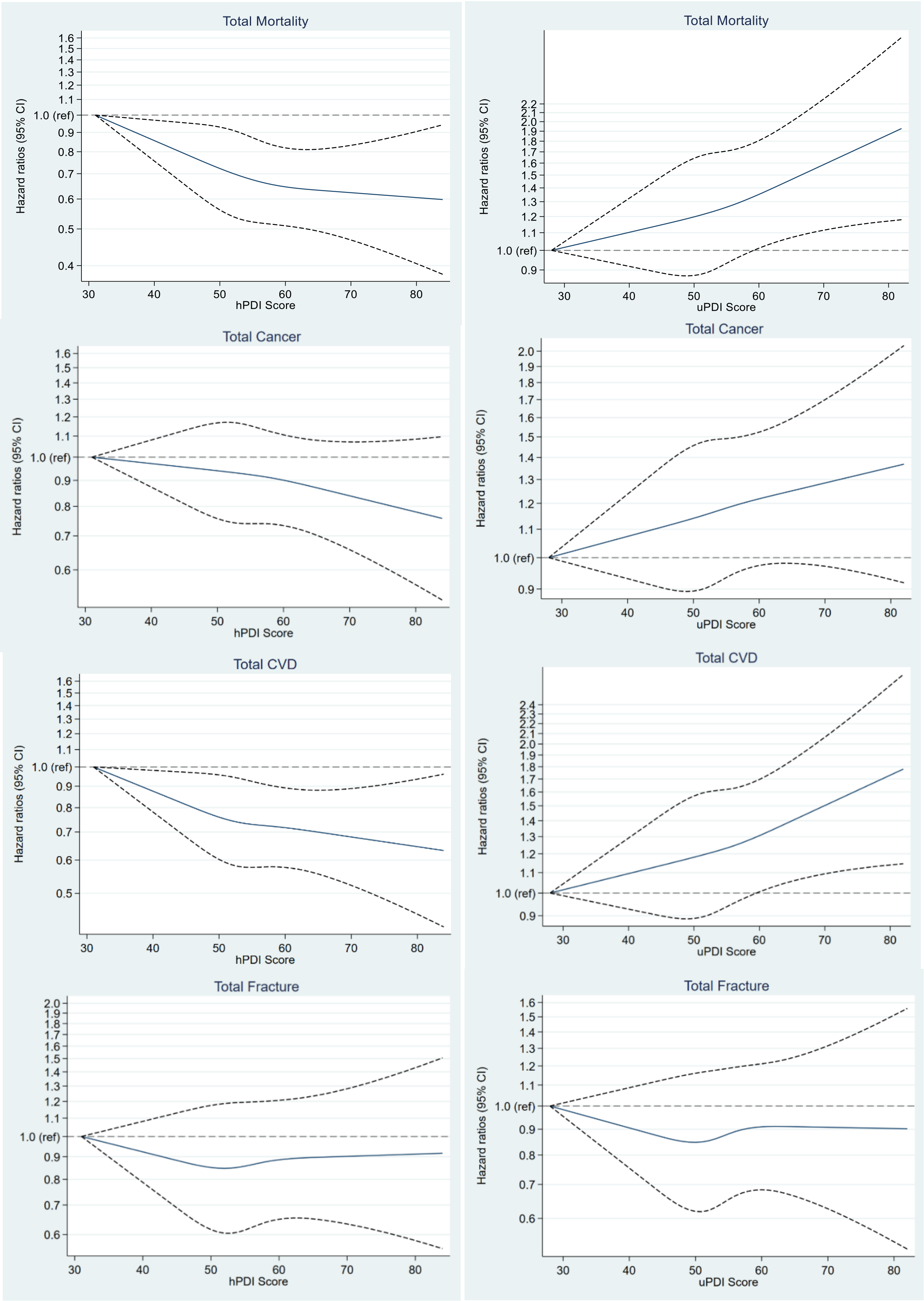

Associations were examined by multivariate Cox regression models with restricted cubic splines of 4 knots. The solid line represents hazard ratio estimates, while the dashed lines represent the 95% confidence intervals (95% CI).

© 2023 Thompson AS et al. *JAMA Network Open*.

eFigure 5. Hazard Ratios (95% CIs) of All-Cause, Cancer, and Cardiovascular Disease Mortality Among UK Biobank Population Subgroups, With Healthful Plant-based Diet Score Modeled as a Continuous Trend (10-Point Increments)

Outcome	Group	Subgroup	N	Cases	HR (95% CI)	p-trend	p-interact
All-Cause Mortality							
	Smoking	Never	72,103	2,460	0.89 (0.83-0.95)	0.001	0.69
		Ever	53,841	3,158	0.85 (0.89-0.99)	<0.001	
	Sex	Male	55,662	3,249	0.87 (0.82-0.93)	<0.001	0.98
		Female	70,555	2,378	0.89 (0.83-0.96)	0.001	
	BMI	Normal	49,508	1,669	0.91 (0.84-0.99)	0.02	0.18
		Overweight	77,426	3,924	0.86 (0.81-0.90)	<0.001	
	Education	Low	52,547	2,241	0.87 (0.81-0.94)	<0.001	0.21
		High	65,079	2,699	0.89 (0.84-0.95)	0.001	
Cancer Mortality							
	Smoking	Never	72,103	1,471	0.94 (0.86-1.03)	0.17	0.95
		Ever	53,841	1,799	0.93 (0.86-1.01)	0.09	
	Sex	Male	55,662	1,707	0.93 (0.86-1.01)	0.09	0.78
		Female	70,555	1,568	0.97 (0.89-1.05)	0.44	
	BMI	Normal	49,508	1,010	0.94 (0.85-1.05)	0.26	0.87
		Overweight	77,426	2,221	0.93 (0.87-1.00)	0.06	
	Education	Low	52,547	1,283	0.94 (0.86-1.04)	0.24	0.15
		High	65,079	1,617	0.96 (0.88-1.04)	0.32	
CVD Mortality							
	Smoking	Never	72,103	303	1.02 (0.84-1.24)	0.85	0.02
		Ever	53,841	395	0.77 (0.65-0.91)	0.002	
	Sex	Male	55,662	449	0.83 (0.71-0.98)	0.02	0.49
		Female	70,555	249	0.99 (0.80-1.22)	0.91	
	BMI	Normal	49,508	190	0.85 (0.66-1.09)	0.19	0.65
		Overweight	77,426	500	0.88 (0.76-1.02)	0.09	
	Education	Low	52,547	286	0.77 (0.62-0.94)	0.01	0.19
		High	65,079	313	0.96 (0.80-1.16)	0.68	

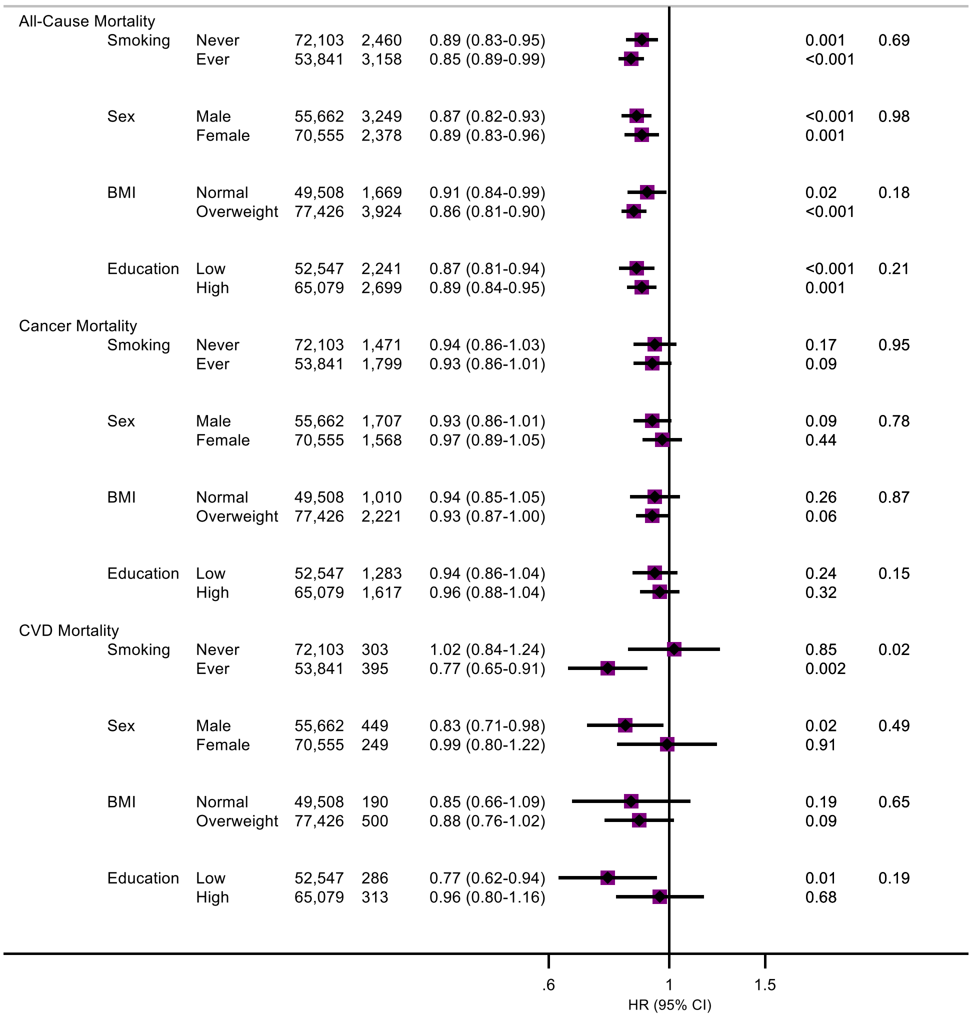

Analyses used age as the underlying time variable and were adjusted for sex (excluding subgroup analysis), BMI (excluding subgroup analysis), ethnicity, physical activity, smoking status (excluding subgroup analysis), alcohol intake, education (excluding subgroup analysis), energy intake, polypharmacy index, multimorbidity index and aspirin use; stratified by region.

For all-cause mortality analyses, models were further adjusted for prevalent CVD and prevalent cancer.

For CVD mortality analyses, models were further adjusted for prevalent CVD.

For cancer mortality analyses, models were further adjusted for prevalent cancer.

Heterogeneity was tested by comparing two models – one without an interaction term between subgroup of interest and hPDI (categorical), with a model that included an interaction term. The likelihood ratio test was used to produce p-interaction values.

Abbreviations: CVD, cardiovascular disease; BMI, Body Mass Index; HR, hazard ratios; CI, confidence intervals.

© 2023 Thompson AS et al. *JAMA Network Open*.

eFigure 6. Hazard Ratios (95% CIs) of Cancer Among UK Biobank Subgroups, With Healthful Plant-based Diet Score Modeled as a Continuous Trend (10-Point Increments)

Outcome	Group	Subgroup	N	Cases	HR (95% CI)	p-trend	p-interact
Any Cancer							
	Smoking	Never	67,462	4,531	0.96 (0.91-1.01)	0.08	0.44
		Ever	49,861	4,392	0.94 (0.89-0.99)	0.01	
	Sex	Male	52,337	4,746	0.95 (0.90-1.00)	0.03	0.78
		Female	65,232	4,193	0.96 (0.91-1.00)	0.10	
	BMI	Normal	67,462	2,912	0.95 (0.88-1.01)	0.10	0.81
		Overweight	71,974	6,012	0.95 (0.91-0.99)	0.02	
	Education	Low	49,184	3,488	0.94 (0.88-0.99)	0.02	0.04
		High	60,528	4,629	0.96 (0.92-1.01)	0.13	
Breast Cancer							
	Smoking	Never	22,442	619	1.07 (0.93-1.23)	0.38	0.25
		Ever	15,347	464	0.84 (0.71-0.98)	0.03	
	BMI	Normal	17,134	410	1.01 (0.85-1.19)	0.94	0.90
		Overweight	20,644	672	0.94 (0.82-1.07)	0.36	
	Education	Low	14,515	413	0.90 (0.76-1.07)	0.24	0.09
		High	20,167	566	0.99 (0.86-1.15)	0.91	
Colorectal Cancer							
	Smoking	Never		461	0.84 (0.72-0.99)	0.03	0.20
		Ever	49,861	498	0.98 (0.84-1.14)	0.78	
	Sex	Male	52,337	562	0.86 (0.74-0.99)	0.03	0.10
		Female	65,232	397	1.00 (0.85-1.20)	0.95	
	BMI	Normal	45,337	280	0.85 (0.69-1.04)	0.11	0.29
		Overweight	71,974	677	0.93 (0.82-1.06)	0.27	
	Education	Low	49,184	385	0.87 (0.73-1.03)	0.11	0.87
		High	60,528	481	0.94 (0.80-1.09)	0.41	
Prostate Cancer							
	Smoking	Never	27,470	1,098	1.03 (0.93-1.14)	0.58	0.17
		Ever	24,747	1,034	0.92 (0.83-1.02)	0.12	
	BMI	Normal	15,192	595	1.01 (0.88-1.16)	0.92	0.76
		Overweight	37,012	1,538	0.97 (0.89-1.06)	0.50	
	Education	Low	22,254	790	0.96 (0.85-1.08)	0.51	0.51
		High	26,462	1,166	0.98 (0.89-1.08)	0.69	

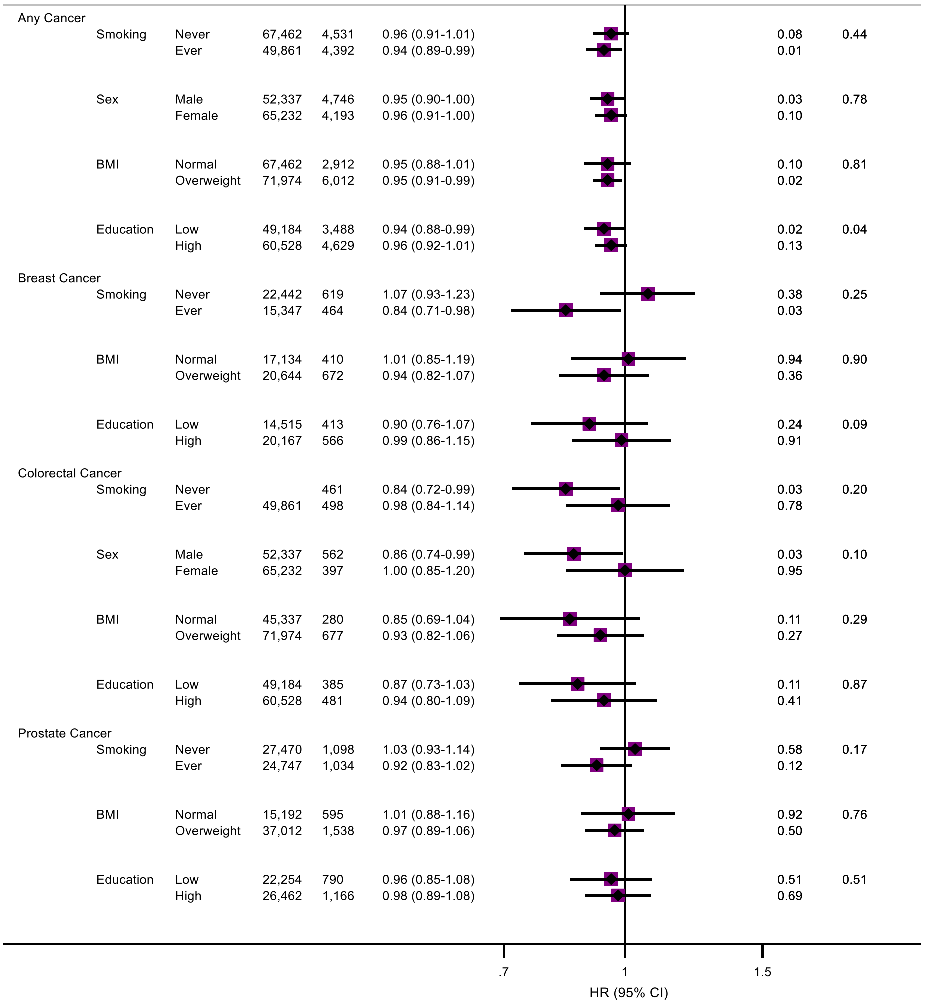

Analyses used age as the underlying time variable and were adjusted for sex (excluding breast and prostate cancer analyses and in subgroup analysis), BMI (excluding subgroup analysis), ethnicity, physical activity, smoking status (excluding subgroup analysis), alcohol intake, education (excluding subgroup analysis), energy intake, polypharmacy index, multimorbidity index and aspirin use; stratified by region.
For any cancer analyses, models were further adjusted for menopausal status and use of MHT.
For breast cancer analyses, models were restricted to postmenopausal breast cancer cases and were further adjusted for use of MHT, use of oral contraception, PRS (BC), age at menarche and age at first live birth.
For colorectal cancer analyses, models were further adjusted for menopausal status, PRS (CRC) and MHT.
For prostate cancer analyses, models were further adjusted for PRS (PC).
Heterogeneity was tested by comparing two models – one without an interaction term between subgroup of interest and hPDI (categorical), with a model that included an interaction term. The likelihood ratio test was used to produce p-interaction values.
Abbreviations: BMI, body mass index; MHT, Menopause hormone therapy; BC, breast cancer; PRS, polygenic risk score; CRC, Colorectal cancer; PC, Prostate Cancer; HR, hazard ratios; CI, confidence intervals.

© 2023 Thompson AS et al. *JAMA Network Open*.

eFigure 7. Hazard Ratios (95% CIs) of Cardiovascular Disease Among UK Biobank Subgroups, With Healthful Plant-based Diet Score Modeled as a Continuous Trend (10-Point Increments)

Outcome	Group	Subgroup	N	Cases	HR (95% CI)	p-trend	p-interact
Any CVD	Smoking	Never	70,751	3,178	0.93 (0.88-0.99)	0.02	0.32
		Ever	52,126	3,693	0.91 (0.86-0.97)	<0.001	
	Sex	Male	53,623	4,528	0.91 (0.87-0.96)	<0.001	0.68
		Female	69,511	2,362	0.97 (0.90-1.04)	0.41	
	BMI	Normal	47,875	1,774	0.96 (0.89-1.04)	0.31	0.68
		Overweight	74,993	5,098	0.91 (0.87-0.96)	<0.001	
	Education	Low	51,350	2,810	0.97 (0.91-1.03)	0.33	0.42
		High	63,652	3,249	0.92 (0.86-0.97)	0.003	
Ischaemic stroke	Smoking	Never	70,751	557	0.84 (0.73-0.97)	0.02	0.22
		Ever	52,126	593	0.94 (0.81-1.08)	0.35	
	Sex	Male	53,623	685	0.84 (0.74-0.96)	0.009	0.26
		Female	69,511	466	0.98 (0.83-1.15)	0.77	
	BMI	Normal	47,875	339	0.91 (0.76-1.10)	0.34	0.88
		Overweight	74,993	807	0.88 (0.78-0.99)	0.04	
	Education	Low	51,350	465	0.98 (0.83-1.15)	0.77	0.02
		High	63,652	565	0.87 (0.75-1.00)	0.06	
Haemorrhagic stroke	Smoking	Never	70,751	208	0.87 (0.69-1.10)	0.26	0.08
		Ever	52,126	261	0.92 (0.75-1.14)	0.44	
	Sex	Male	53,623	282	0.94 (0.77-1.14)	0.53	0.67
		Female	69,511	187	0.87 (0.67-1.11)	0.27	
	BMI	Normal	47,875	151	0.97 (0.74-1.28)	0.85	0.04
		Overweight	74,993	316	0.88 (0.73-1.06)	0.18	
	Education	Low	51,350	184	0.98 (0.76-1.26)	0.87	0.86
		High	63,652	227	0.89 (0.72-1.11)	0.30	
MI	Smoking	Never	70,751	1,349	0.92 (0.84-1.01)	0.08	0.53
		Ever	52,126	1,897	0.87 (0.80-0.94)	<0.001	
	Sex	Male	53,623	2,383	0.90 (0.84-0.96)	0.002	0.82
		Female	69,511	870	0.92 (0.82-1.03)	0.16	
	BMI	Normal	47,875	758	0.95 (0.84-1.07)	0.37	0.61
		Overweight	74,993	2,485	0.89 (0.83-0.95)	0.001	
	Education	Low	51,350	1,346	0.95 (0.86-1.04)	0.25	0.82
		High	63,652	1,474	0.90 (0.82-0.98)	0.01	

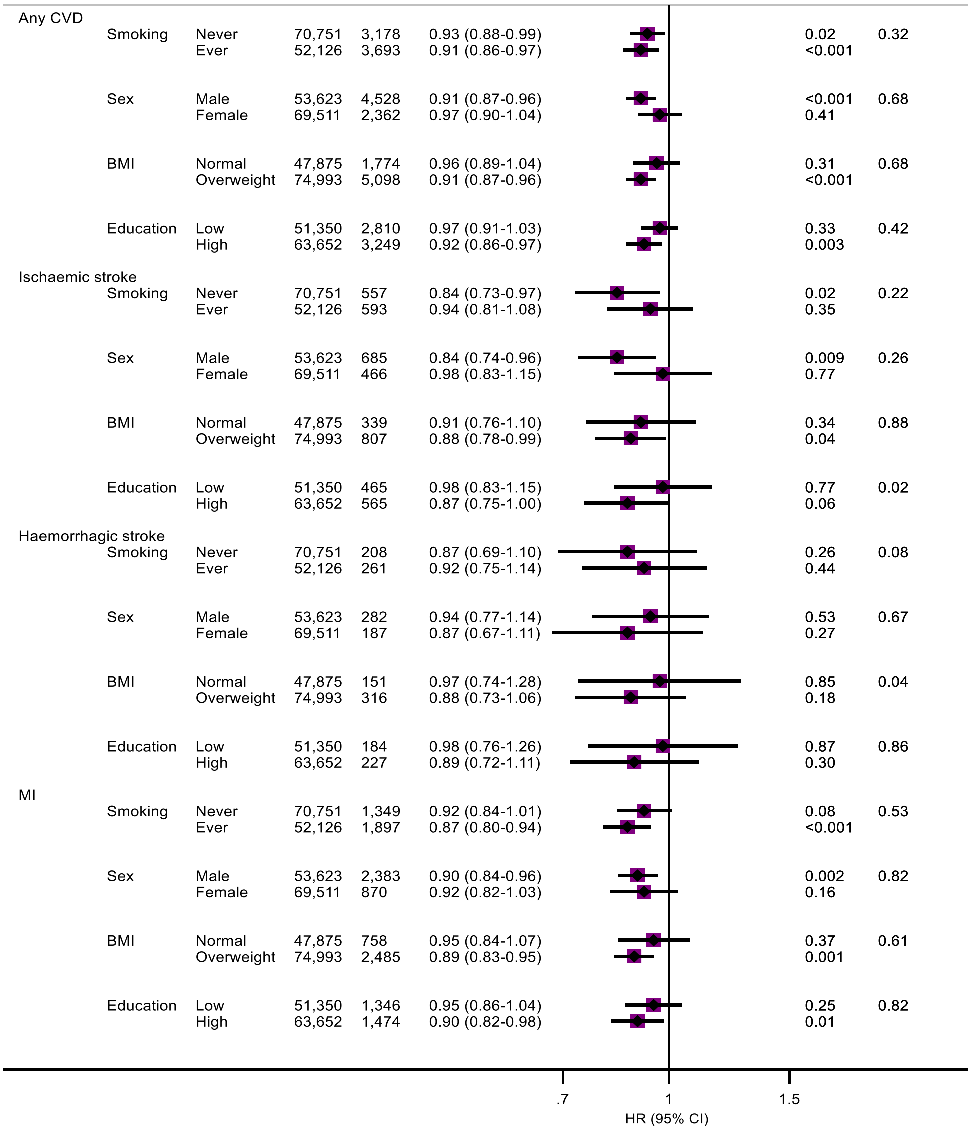

Analyses used age as the underlying time variable and were adjusted for sex (excluding subgroup analysis), BMI (excluding subgroup analysis), ethnicity, physical activity, smoking status (excluding subgroup analysis), alcohol intake, education (excluding subgroup analyses), energy intake, polypharmacy index, multimorbidity index and aspirin use; stratified by region.
For any CVD and haemorrhagic stroke, models were further adjusted for PRS (CVD).
For ischaemic stroke analyses, models were further adjusted for PRS (IS).
For MI, models were further adjusted for PRS (CAD).
Heterogeneity was tested by comparing two models – one without an interaction term between subgroup of interest and hPDI (categorical), with a model that included an interaction term. The likelihood ratio test was used to produce p-interaction values.
Abbreviation: BMI, Body Mass Index; CVD, cardiovascular disease; PRS, polygenic risk score; IS, Ischaemic stroke; MI, myocardial infarction; CAD, coronary artery disease; HR, hazard ratios; CI, confidence intervals.

© 2023 Thompson AS et al. *JAMA Network Open*.

eFigure 8. Hazard Ratios (95% CIs) of Fracture Among UK Biobank Subgroups, With Healthful Plant-based Diet Score Modeled as a Continuous Trend (10-Point Increments)

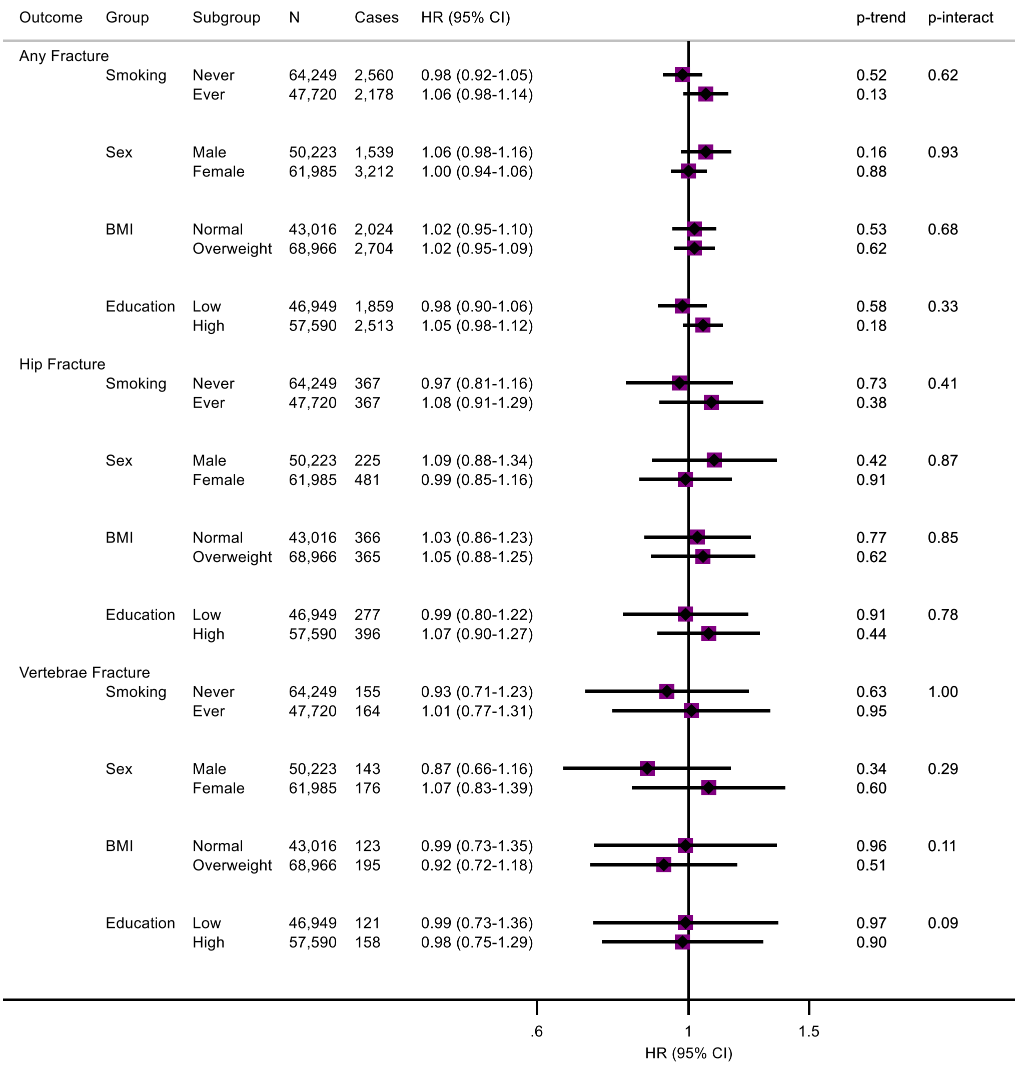

Outcome	Group	Subgroup	N	Cases	HR (95% CI)	p-trend	p-interact
Any Fracture							
	Smoking	Never	64,249	2,560	0.98 (0.92-1.05)	0.52	0.62
		Ever	47,720	2,178	1.06 (0.98-1.14)	0.13	
	Sex	Male	50,223	1,539	1.06 (0.98-1.16)	0.16	0.93
		Female	61,985	3,212	1.00 (0.94-1.06)	0.88	
	BMI	Normal	43,016	2,024	1.02 (0.95-1.10)	0.53	0.68
		Overweight	68,966	2,704	1.02 (0.95-1.09)	0.62	
	Education	Low	46,949	1,859	0.98 (0.90-1.06)	0.58	0.33
		High	57,590	2,513	1.05 (0.98-1.12)	0.18	
Hip Fracture							
	Smoking	Never	64,249	367	0.97 (0.81-1.16)	0.73	0.41
		Ever	47,720	367	1.08 (0.91-1.29)	0.38	
	Sex	Male	50,223	225	1.09 (0.88-1.34)	0.42	0.87
		Female	61,985	481	0.99 (0.85-1.16)	0.91	
	BMI	Normal	43,016	366	1.03 (0.86-1.23)	0.77	0.85
		Overweight	68,966	365	1.05 (0.88-1.25)	0.62	
	Education	Low	46,949	277	0.99 (0.80-1.22)	0.91	0.78
		High	57,590	396	1.07 (0.90-1.27)	0.44	
Vertebrae Fracture							
	Smoking	Never	64,249	155	0.93 (0.71-1.23)	0.63	1.00
		Ever	47,720	164	1.01 (0.77-1.31)	0.95	
	Sex	Male	50,223	143	0.87 (0.66-1.16)	0.34	0.29
		Female	61,985	176	1.07 (0.83-1.39)	0.60	
	BMI	Normal	43,016	123	0.99 (0.73-1.35)	0.96	0.11
		Overweight	68,966	195	0.92 (0.72-1.18)	0.51	
	Education	Low	46,949	121	0.99 (0.73-1.36)	0.97	0.09
		High	57,590	158	0.98 (0.75-1.29)	0.90	

Analyses used age as the underlying time variable and were adjusted for sex (excluding subgroup analysis), BMI (excluding subgroup analysis), ethnicity, physical activity, smoking status (excluding subgroup analysis), alcohol intake, education (excluding subgroup analyses), energy intake, vitamin/mineral supplement use, PRS (OP), polypharmacy index, multimorbidity index and aspirin use; stratified by region.
Heterogeneity was tested by comparing two models – one without an interaction term between subgroup of interest and hPDI (categorical), with a model that included an interaction term. The likelihood ratio test was used to produce p-interaction values.
Abbreviations: BMI, Body Mass Index; PRS, polygenic risk score; OP, osteoporosis; HR, hazard ratios; CI, confidence intervals.

© 2023 Thompson AS et al. *JAMA Network Open*.

eFigure 9. Hazard Ratios (95% CIs) of Cancer Across Strata of Genetic Cancer Risk, With Healthful Plant-based Diet Score Modeled as a Continuous Trend (10-Point Increments)

Outcome	PRS	N	Cases	HR (95% CI)	p-trend	p-interact
Breast Cancer						
	Low	12,465	185	0.82 (0.64-1.06)	0.14	0.74
	Medium	12,399	306	0.99 (0.81-1.21)	0.90	
	High	12,078	563	1.01 (0.87-1.16)	0.94	
Colorectal Cancer						
	Low	38,357	174	0.93 (0.72-1.20)	0.57	0.64
	Medium	38,357	296	0.99 (0.82-1.20)	0.94	
	High	38,356	466	0.87 (0.75-1.02)	0.10	
Prostate Cancer						
	Low	17,128	237	0.92 (0.74-1.14)	0.44	0.35
	Medium	17,127	589	1.05 (0.91-1.20)	0.52	
	High	17,127	1,272	0.96 (0.87-1.05)	0.39	

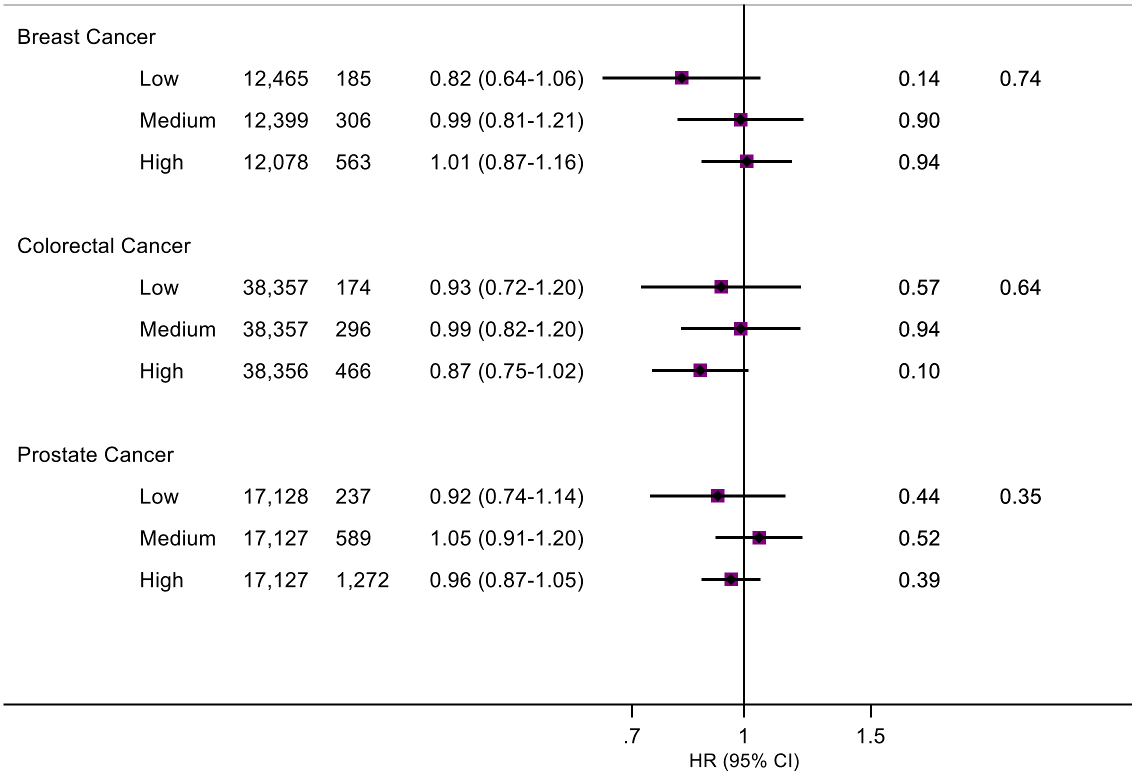

PRS were obtained from the UK Biobank Showcase for breast, bowel, and prostate cancer.
Analyses used age as the underlying time variable and were adjusted for sex (excluding breast and prostate cancer analyses), BMI, ethnicity, physical activity, smoking status, alcohol intake, education, energy intake, polypharmacy index, multimorbidity index and aspirin use; stratified by region.
For breast cancer analyses, models were restricted to postmenopausal breast cancer cases and were further adjusted for use of MHT, use of oral contraception, age at menarche and age at first live birth.
For colorectal cancer analyses, models were further adjusted for menopausal status, and MHT.
Heterogeneity was tested by comparing two models – one without an interaction term between subgroup of interest and hPDI (categorical), with a model that included an interaction term. The likelihood ratio test was used to produce p-interaction values.
Abbreviations: BMI, Body Mass Index; PRS, polygenic risk score; MHT, Menopause hormone therapy; HR, hazard ratios; CI, confidence intervals.

© 2023 Thompson AS et al. *JAMA Network Open*.

eFigure 10. Hazard Ratios (95% CIs) of Fracture Across Strata of Genetic Osteoporosis Risk, With Healthful Plant-based Diet Score Modeled as a Continuous Trend (10-Point Increments)

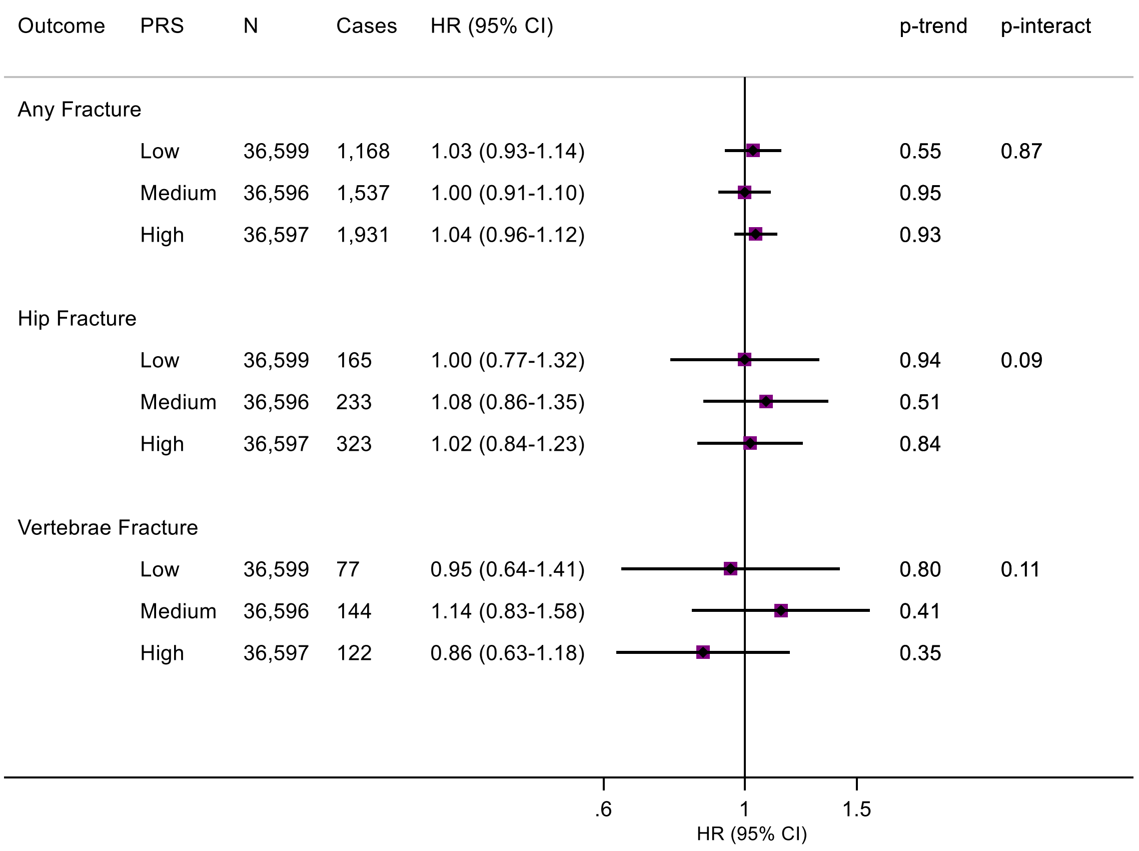

PRS were obtained from the UK Biobank Showcase for OP.

Analyses used age as the underlying time variable and were adjusted for sex, BMI, ethnicity, physical activity, smoking status, alcohol intake, education, energy intake, vitamin/mineral supplement use, polypharmacy index, multimorbidity index and aspirin use; stratified by region.

Heterogeneity was tested by comparing two models – one without an interaction term between subgroup of interest and hPDI (categorical), with a model that included an interaction term. The likelihood ratio test was used to produce p-interaction values.

Abbreviations: PRS, polygenic risk score; OP, Osteoporosis; BMI, Body Mass Index; HR, hazard ratios; CI, confidence intervals.

© 2023 Thompson AS et al. *JAMA Network Open*.

eFigure 11. Sensitivity Analyses Showing Hazard Ratios (95% CIs) Across Sex-Specific Healthful vs Unhealthful Plant-based Diet Index Quartiles, Removing the First 2 Years of Follow-up for Participants Who Completed 2 or More Dietary Assessments and the Associated Risk of All-Cause, Cancer, and Cardiovascular Disease Mortality

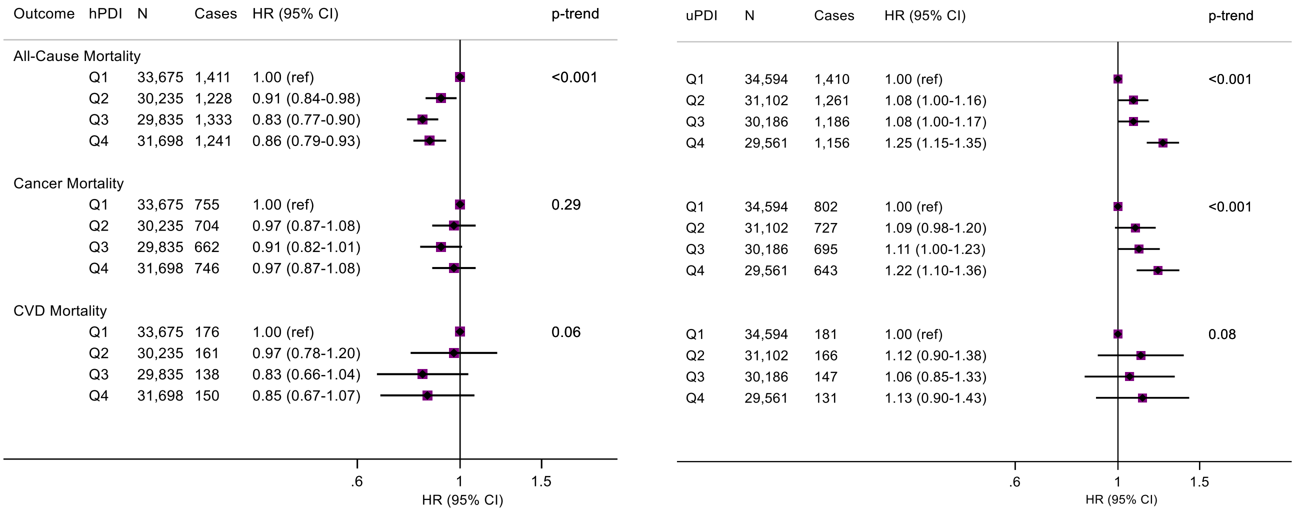

Analyses used age as the underlying time variable and were adjusted for sex, BMI, ethnicity, physical activity, smoking status, alcohol intake, education, energy intake, polypharmacy index, multimorbidity index and aspirin use; stratified by region.

For all-cause mortality analyses, models were further adjusted for prevalent CVD and prevalent cancer.

For CVD mortality analyses, models were further adjusted for prevalent CVD.

For cancer mortality analyses, models were further adjusted for prevalent cancer.

Abbreviations: hPDI, healthful plant-based diet index; uPDI, unhealthful plant-based diet index; CVD, cardiovascular disease; BMI, Body Mass Index HR, hazard ratios; CI, confidence intervals.

© 2023 Thompson AS et al. *JAMA Network Open*.

eFigure 12. Sensitivity Analyses Showing Hazard Ratios (95% CIs) Across Sex-Specific Healthful vs Unhealthful Plant-based Diet Index Quartiles, Removing the First 2 Years of Follow-up for Participants Who Completed 2 or More Dietary Assessments and the Associated Risk of Any Cancer, Postmenopausal Breast Cancer, Colorectal Cancer, and Prostate Cancer

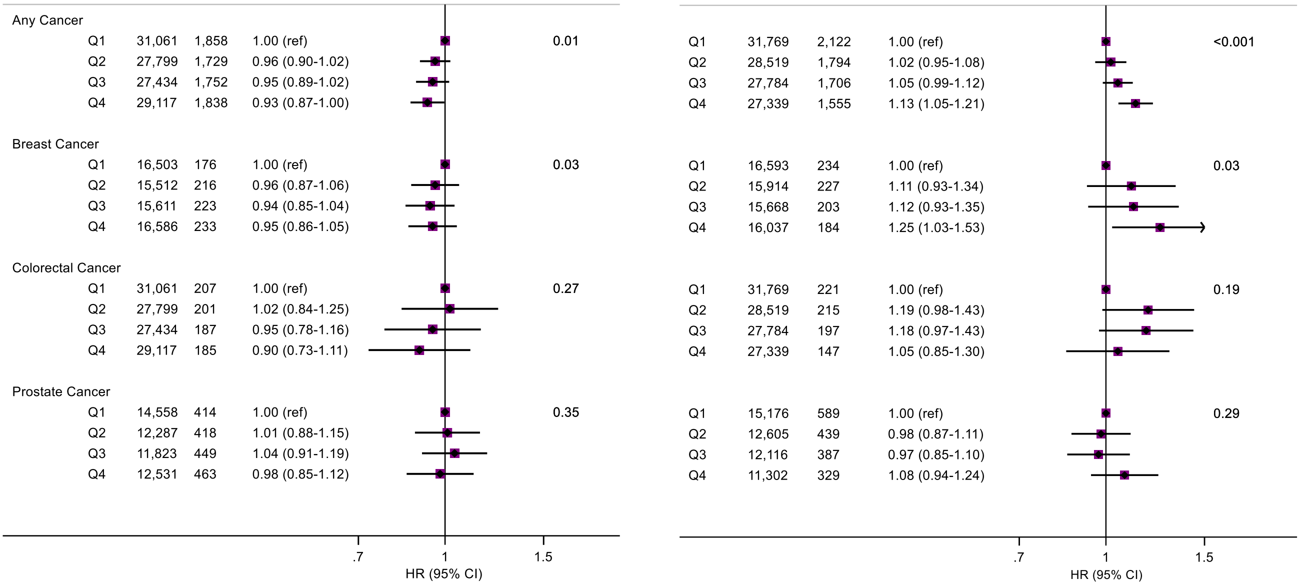

Outcome	hPDI	N	Cases	HR (95% CI)	p-trend
Any Cancer					
	Q1	31,061	1,858	1.00 (ref)	0.01
	Q2	27,799	1,729	0.96 (0.90-1.02)	
	Q3	27,434	1,752	0.95 (0.89-1.02)	
	Q4	29,117	1,838	0.93 (0.87-1.00)	
Breast Cancer					
	Q1	16,503	176	1.00 (ref)	0.03
	Q2	15,512	216	0.96 (0.87-1.06)	
	Q3	15,611	223	0.94 (0.85-1.04)	
	Q4	16,586	233	0.95 (0.86-1.05)	
Colorectal Cancer					
	Q1	31,061	207	1.00 (ref)	0.27
	Q2	27,799	201	1.02 (0.84-1.25)	
	Q3	27,434	187	0.95 (0.78-1.16)	
	Q4	29,117	185	0.90 (0.73-1.11)	
Prostate Cancer					
	Q1	14,558	414	1.00 (ref)	0.35
	Q2	12,287	418	1.01 (0.88-1.15)	
	Q3	11,823	449	1.04 (0.91-1.19)	
	Q4	12,531	463	0.98 (0.85-1.12)	

uPDI	N	Cases	HR (95% CI)	p-trend
Q1	31,769	2,122	1.00 (ref)	<0.001
Q2	28,519	1,794	1.02 (0.95-1.08)	
Q3	27,784	1,706	1.05 (0.99-1.12)	
Q4	27,339	1,555	1.13 (1.05-1.21)	
Q1	16,593	234	1.00 (ref)	0.03
Q2	15,914	227	1.11 (0.93-1.34)	
Q3	15,668	203	1.12 (0.93-1.35)	
Q4	16,037	184	1.25 (1.03-1.53)	
Q1	31,769	221	1.00 (ref)	0.19
Q2	28,519	215	1.19 (0.98-1.43)	
Q3	27,784	197	1.18 (0.97-1.43)	
Q4	27,339	147	1.05 (0.85-1.30)	
Q1	15,176	589	1.00 (ref)	0.29
Q2	12,605	439	0.98 (0.87-1.11)	
Q3	12,116	387	0.97 (0.85-1.10)	
Q4	11,302	329	1.08 (0.94-1.24)	

Analyses use age as the underlying time variable and were adjusted for adjusted for sex (excluding breast and prostate cancer analyses), BMI, ethnicity, physical activity, smoking status, alcohol intake, education, energy intake, polypharmacy index, multimorbidity index and aspirin use; stratified by region.
For any cancer analyses, models were further adjusted for menopausal status and use of MHT.
For breast cancer analyses, models were restricted to postmenopausal breast cancer cases and were further adjusted for use of MHT, use of oral contraception, PRS (BC), age at menarche and age at first live birth.
For colorectal cancer analyses, models were further adjusted for menopausal status, PRS (CRC) and MHT.
For prostate cancer analyses, models were further adjusted for PRS (PC).
Abbreviations: hPDI, healthful plant-based diet index; uPDI, unhealthful plant-based diet index; BMI, Body Mass Index; MHT, Menopause hormone therapy; PRS, polygenic risk score; BC, breast cancer; CRC, Colorectal cancer; PC, Prostate Cancer; HR, hazard ratios; CI, confidence intervals.

© 2023 Thompson AS et al. *JAMA Network Open*.

eFigure 13. Sensitivity Analyses Showing Hazard Ratios (95% CIs) Across Sex-Specific Healthful vs Unhealthful Plant-based Diet Index Quartiles, Removing the First 2 Years of Follow-up for Participants Who Completed 2 or More Dietary Assessments and the Associated Risk of Any Cardiovascular Disease, Ischemic Stroke, Hemorrhagic Stroke, and Myocardial Infarction

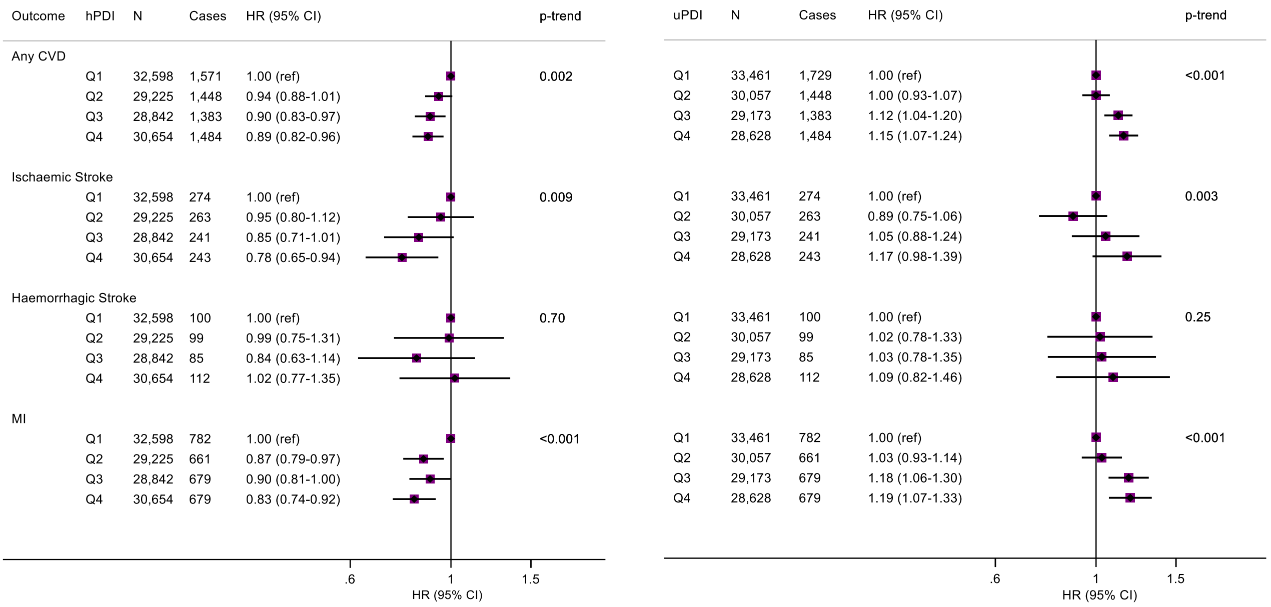

Analyses used age as the underlying time variable and were adjusted for sex, BMI, ethnicity, physical activity, smoking status, alcohol intake, education, energy intake, polypharmacy index, multimorbidity index and aspirin use; stratified by region.
For any CVD and haemorrhagic stroke analyses, models were further adjusted for PRS (CVD).
For ischaemic stroke analyses, models were further adjusted for PRS (IS).
For MI analyses, models were further adjusted for PRS (CAD).
Abbreviations: hPDI, healthful plant-based diet index; uPDI, unhealthful plant-based diet index; CVD, cardiovascular disease; MI, myocardial infarction; BMI, Body Mass Index; PRS, polygenic risk score; IS, Ischaemic stroke; CAD, coronary artery disease; HR, hazard ratios; CI, confidence intervals.

© 2023 Thompson AS et al. *JAMA Network Open*.

eFigure 14. Sensitivity Analyses Showing Hazard Ratios (95% CIs) Across Sex-Specific Healthful vs Unhealthful Plant-based Diet Index Quartiles, Removing the First 2 Years of Follow-up for Participants Who Completed 2 or More Dietary Assessments and the Associated Risk of Any Fracture, Hip Fracture, and Vertebrae Fracture

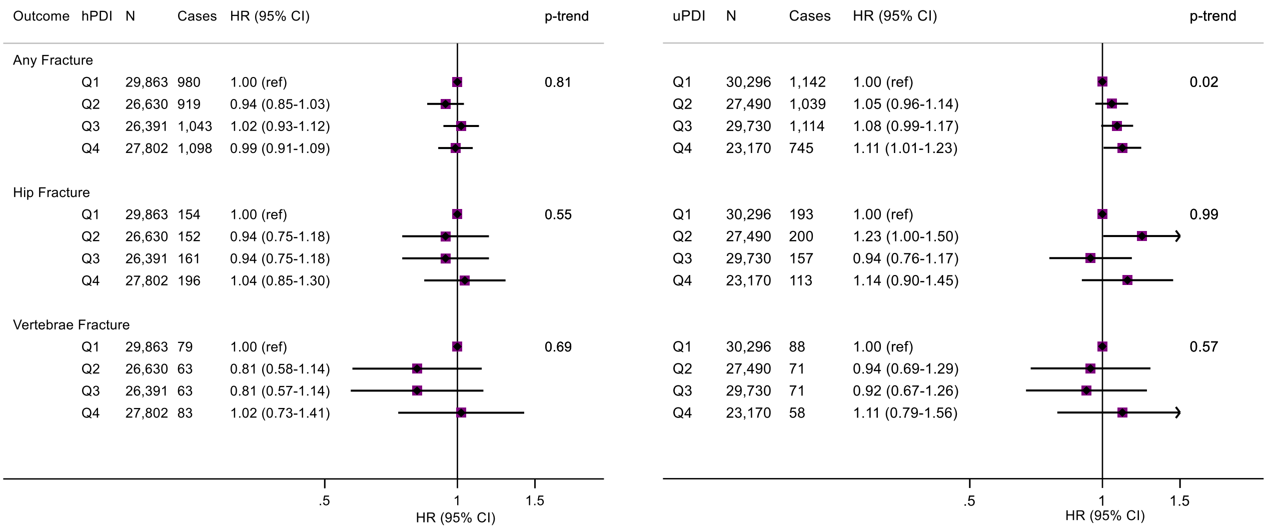

Analyses used age as the underlying time variable and were adjusted for sex, BMI, ethnicity, physical activity, smoking status, alcohol intake, education, energy intake, vitamin/mineral supplement use, PRS (OP), polypharmacy index, multimorbidity index and aspirin use; stratified by region.

Abbreviations: hPDI, healthful plant-based diet index; uPDI, unhealthful plant-based diet index; BMI, Body Mass Index; PRS, polygenic risk score; OP, osteoporosis; HR, hazard ratios; CI, confidence intervals.

© 2023 Thompson AS et al. *JAMA Network Open*.

eFigure 15. Multivariable-Adjusted Hazard Ratios (95% CIs) of All-Cause Mortality (n = 126 217), Cancer (n = 117 569), Cardiovascular Disease (n = 123 134), and Fracture (n = 112 208) Among Ethnic Subgroups Across Sex-Specific Healthful vs Unhealthful Plant-based Diet Index Quartiles

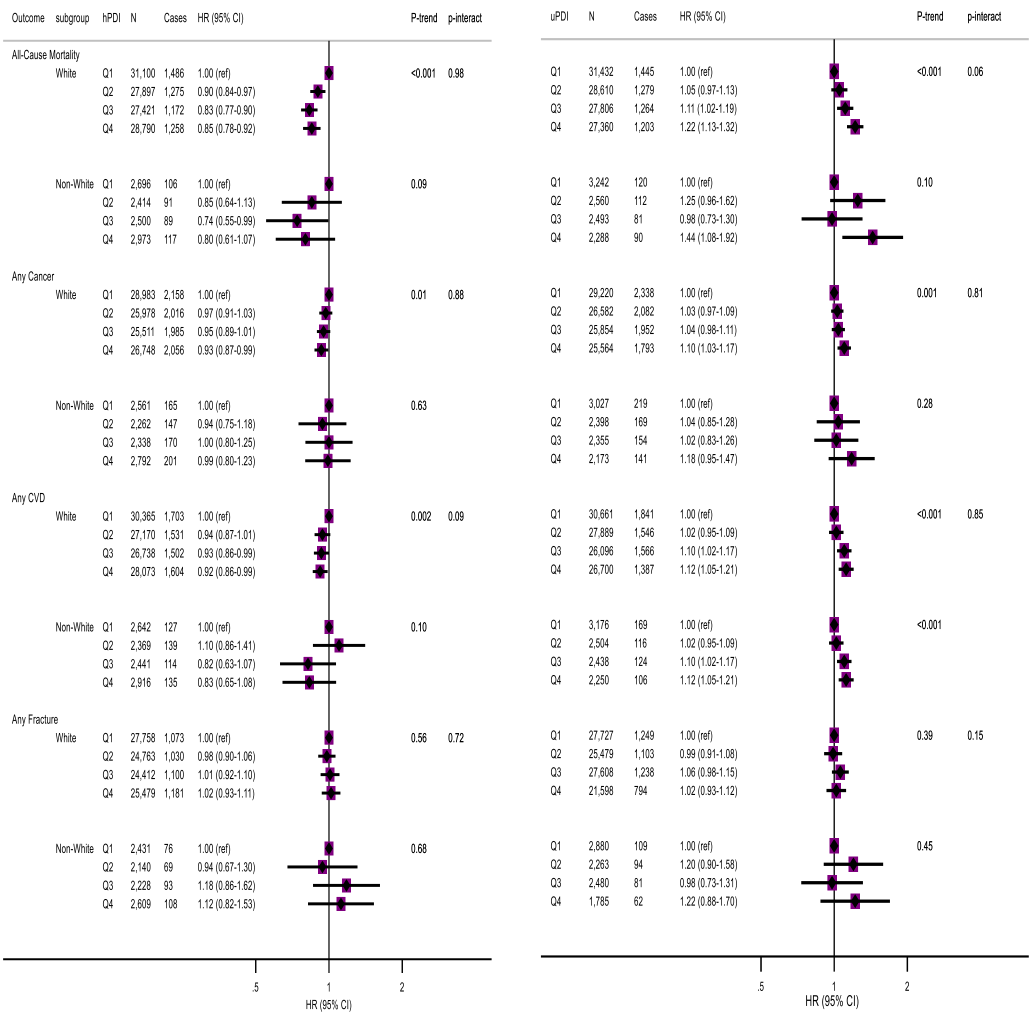

All models used age as the underlying time variable and were adjusted for sex, BMI, physical activity, smoking status, alcohol intake, education, energy intake, polypharmacy index, multimorbidity index and aspirin use; stratified by region.

For all-cause mortality analyses, models were further adjusted for prevalent CVD and prevalent cancer.

For any cancer analyses, models were further adjusted for menopausal status and use of MHT.

For any CVD analyses, models were further adjusted for PRS (CVD).

For any fracture analyses, models further adjusted for vitamin/mineral supplement use and PRS (OP).

P-trend is for linear trend.

Heterogeneity was tested by comparing two models – one without an interaction term between subgroup of interest and hPDI (categorical), with a model that included an interaction term. The likelihood ratio test was used to produce p-interaction values.

Abbreviations: Q, quartile; hPDI, healthful plant-based diet index; uPDI, unhealthful plant-based diet index; CVD, Cardiovascular Disease; BMI, body mass index; PRS, polygenic risk score; MHT, menopause hormone therapy; OP, Osteoporosis; HR, hazard ratios; CI, confidence intervals.

© 2023 Thompson AS et al. *JAMA Network Open*.

eReferences

1. Willett WC, Howe GR, Kushi LH. Adjustment for total energy intake in epidemiologic studies. *The American Journal of Clinical Nutrition.* 1997;65(4):1220S-1228S.
2. Masnoon N, Shakib S, Kalisch-Ellett L, Caughey GE. What is polypharmacy? A systematic review of definitions. *BMC geriatrics.* 2017;17(1):1-10.
3. Thompson DJ, Wells D, Selzam S, et al. UK Biobank release and systematic evaluation of optimised polygenic risk scores for 53 diseases and quantitative traits. *medRxiv.* 2022:2022.2006.2016.22276246.
4. Bradbury KE, Young HJ, Guo W, Key TJ. Dietary assessment in UK Biobank: an evaluation of the performance of the touchscreen dietary questionnaire. *J Nutr Sci.* 2018;7:e6-e6.
5. Liu B, Young H, Crowe FL, et al. Development and evaluation of the Oxford WebQ, a low-cost, web-based method for assessment of previous 24 h dietary intakes in large-scale prospective studies. *Public Health Nutr.* 2011;14(11):1998-2005.
6. Greenwood DC, Hardie LJ, Frost GS, et al. Validation of the Oxford WebQ Online 24-Hour Dietary Questionnaire Using Biomarkers. *Am J Epidemiol.* 2019;188(10):1858-1867.
7. Galante J, Adamska L, Young A, et al. The acceptability of repeat Internet-based hybrid diet assessment of previous 24-h dietary intake: administration of the Oxford WebQ in UK Biobank. *British Journal of Nutrition.* 2016;115(4):681-686.
8. Sudlow C, Gallacher J, Allen N, et al. UK biobank: an open access resource for identifying the causes of a wide range of complex diseases of middle and old age. *PLoS medicine.* 2015;12(3):e1001779-e1001779.
9. Warriner AH, Patkar NM, Curtis JR, et al. Which fractures are most attributable to osteoporosis? *J Clin Epidemiol.* 2011;64(1):46-53.
10. Mei Z, Chen G-C, Hu J, et al. Habitual use of fish oil supplements, genetic predisposition, and risk of fractures: a large population-based study. *The American Journal of Clinical Nutrition.* 2021;114(3):945-954.

© 2023 Thompson AS et al. *JAMA Network Open*.
