# Supplementary material for: Association of Healthful Plant-based Diet Adherence With Risk of Mortality and Major Chronic Diseases Among Adults in the UK
Source: JAMA Netw Open. 2023 Mar 28;6(3):e234714. doi: 10.1001/jamanetworkopen.2023.4714 (PMC10051114; doi:10.1001/jamanetworkopen.2023.4714)
Supplement: Supplement 2. — Data Sharing Statement [file jamanetwopen-e234714-s002.pdf]

Data Sharing Statement

Thompson. Association of Healthful Plant-based Diet Adherence With Risk of Mortality and Major Chronic Diseases Among Adults in the UK. *JAMA Netw Open*. Published March 28, 2023. doi:10.1001/jamanetworkopen.2023.4714

Data
Data available: No

Additional Information
Explanation for why data not available: Data from the UK Biobank cohort was used for the present study. A publication of the dataset in public repositories is not possible for data protection reasons, but the analytical dataset will be made available by the corresponding author upon request.
